# Tracking Structural and Electron Spin Density Changes in a Cooperative Mn^3+^ Spin Crossover Complex at Atomic Scale via Low Temperature Solid‐State NMR

**DOI:** 10.1002/anie.202517466

**Published:** 2025-12-10

**Authors:** Wassilios Papawassiliou, José P. Carvalho, Subhradip Paul, Aizuddin Sultan, Michael Fardis, Georgios Papavassiliou, Grace G. Morgan, Katharina Märker, Gaël De Paëpe

**Affiliations:** ^1^ Univ Grenoble Alpes, CEA, IRIG, MEM Grenoble 38000 France; ^2^ Interdisciplinary Nanoscience Center (iNANO) and Department of Chemistry Aarhus University Gustav Wieds Vej 14 Aarhus C DK‐8000 Denmark; ^3^ School of Chemistry University College Dublin Belfield Dublin 4 Ireland; ^4^ Institute of Nanoscience and Nanotechnology NCSR “DEMOKRITOS” 153 41 Ag. Paraskevi – Attiki Athens Greece

**Keywords:** First principle calculations, Solid‐state NMR, Spin crossover, Transition metal complexes

## Abstract

Electron spin‐state changes in transition‐metal (TM) complexes underpin many biochemical processes and molecular spin‐control technologies. Such transitions, triggered by external stimuli like temperature, light, or pressure, alter both the molecular structure and electron spin density (ESD) distribution. Paramagnetic NMR offers atomic‐scale insights into these changes, yet traditional solution‐state measurements bear limitations due to solvent effects, unaccounted lattice cooperativity, and inaccessibility at cryogenic temperatures. We overcome these limitations by extending the approach to spinning solids at cryogenic temperatures. Specifically, we report high‐resolution ^13^C and ^1^H magic‐angle spinning (MAS) NMR spectra of a mononuclear spin‐crossover (SCO) Mn(III) complex across the SCO transition at 130 K. Such low‐temperature experiments are particularly challenging because paramagnetic shift and shift anisotropy are inversely proportional to the temperature. The experimental findings are supported by advanced quantum chemical calculations of the NMR and EPR parameters to assign and rationalize the observed paramagnetic shifts. Additionally, monitoring selected ^1^H resonances upon heating and cooling through the transition provides access to the order parameter ($q=\frac{N_{\mathrm{HS}}-N_{\mathrm{LS}}}{N_{\mathrm{HS}}+N_{\mathrm{LS}}}$), revealing hysteresis behavior similar to the magnetic susceptibility measurements. This work demonstrates that paramagnetic NMR combined with quantum chemical calculations provides a unique route to probing SCO at the atomic level.

## Graphical abstract

The spin transition of a Mn(III) complex is investigated by tracking the evolution of the ^1^H and ^13^C NMR signals of individual sites under fast low‐temperature magic angle spinning conditions. The proposed experimental methodology combined with ab initio calculations of the paramagnetic NMR shifts provides a unique toolbox to study the electronic structure and dynamics of spin‐crossover systems at cryogenic temperatures.

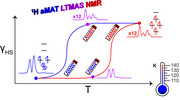

## Introduction

Electrons are fermions with spin *S* = 1/2, subjected to the Pauli exclusion principle; the latter, together with Hund's rule, dictates the Aufbau (filling) of the electron energy states in polyelectronic atoms. In the case of Jahn Teller (JT) active TM complexes, the arrangement of the d‐electrons of the TM is determined by the interplay between these two prior conditions with the JT effect, which, if active, induces bond‐length changes and electron spin density (ESD) redistribution across the molecule. One important example of this effect is the molecular and spin reorganization that occurs in the Mn_4_CaO_5_ cluster during natural photosynthesis. In this process, the cluster adopts a JT‐distorted dark‐stable S1 state as part of Kok's water oxidation cycle.^[^ ^1^, ^2^ ^]^ Another example is the transition in hemoglobin from a low‐spin state (LS, *S* = 1/2) when it is hexacoordinated to a high‐spin state (HS, *S* = 2) when it becomes pentacoordinated in deoxy‐myoglobin. This high‐spin form is pseudo‐JT active and occurs during the respiratory cycle.^[^ ^3^, ^4^ ^]^

In this prospect, significant efforts have been focused on the study of the spin‐crossover (SCO) molecules, which undergo a reversible transition from an LS to an HS electron state, characterized by a hysteresis loop, promoting a memory effect that is critical in molecular spintronic technologies.^[^ ^5^, ^6^ ^]^ The transition is encoded in the evolution of the order parameter^[^ ^7^, ^8^ ^]^ $q=\frac{N_{\mathrm{HS}}-N_{\mathrm{LS}}}{N_{\mathrm{HS}}+N_{\mathrm{LS}}}$, with *N* being the number of paramagnetic centers in the respective spin state; hence, the SCO effect is predominantly observed in first‐row transition metal ion complexes with open d^4^–d^7^ orbitals in a pseudo‐octahedral coordination.^[^ ^9^ ^]^ To date, a substantial body of work featuring Fe(II) and Co(II) complexes has been published, focusing on achieving hysteresis loops with various external stimuli,^[^ ^10^ ^]^ primarily temperature changes.^[^ ^10^, ^11^, ^12^, ^13^, ^14^ ^]^ More recently, Mn(III) complexes have been gaining significant attention^[^ ^15^, ^16^, ^17^, ^18^ ^]^ due to the pronounced JT effect characterizing the high‐spin (HS) *S* = 2 d^4^ state of the Mn(III) center. In such systems, the JT effect induces a strong equatorial bond length stretching, consequently affecting the SCO transition. In this context, flexible ligands coordinated to the Mn(III) center are a prerequisite for the appearance of the SCO effect, as they allow electrons to occupy the $d_{x^{2}-y^{2}}$ orbitals along the *xy*‐plane.^[^ ^18^, ^19^ ^]^

The current experimental toolbox for probing spin‐crossover (SCO) molecules near their spin transition encompasses both macroscopic and microscopic approaches. Macroscopic methods, such as magnetic susceptibility measurements and variable‐temperature X‐ray diffraction, capture collective changes in the electronic or crystal structure, respectively. In contrast, common microscopic techniques are variable‐temperature electron paramagnetic resonance (EPR) and Raman spectroscopy, which respectively monitor local changes at the metal center and vibrational fingerprints associated with the transition. Their applicability, however, depends strongly on the system under study: in Fe(II) SCO complexes, for instance, the diamagnetic low‐spin state is EPR silent (*S*  =  0), while in Mn(III) systems the low‐spin state (*S* = 1) can only be accessed by high‐frequency EPR at cryogenic temperatures.^[^ ^15^ ^]^ Raman spectroscopy, on the other hand, provides only indirect evidence of spin‐state switching, determined from variations in metal─ligand bond vibrations.^[^ ^18^ ^]^

NMR spectroscopy at variable temperatures stands out as an experimental technique that provides atomic‐scale insights into both the structural and the electronic changes that take place during the SCO transition.^[^ ^20^, ^21^, ^22^ ^]^ In NMR experiments on SCO molecules, the additional ESD due to unpaired electrons delocalized onto the nuclei of a paramagnetic compound (i) introduces a paramagnetic shift that is reflected in the NMR spectrum compared to its diamagnetic analogue,^[^ ^23^ ^]^ and (ii) induces a paramagnetic relaxation enhancement that allows improving the signal‐to‐noise ratio through the accumulation of a large number of transients but also leads to shortened coherence lifetimes and to broadening of the NMR linewidths.^[^ ^24^, ^25^ ^]^ The paramagnetic shift follows the Curie Law, meaning that it has a linear dependence with respect to the inverse of the temperature (in absence of spin‐orbit coupling effects),^[^ ^25^ ^]^ and each slope depends on the strength of the hyperfine coupling to the paramagnetic center.

To this date, NMR on SCO molecules has been employed primarily in solution and more specifically on molecules that undergo a spin transition at operational temperatures of the relevant NMR probes (i.e., 188–423 K).^[^ ^20^, ^21^, ^23^, ^26^, ^27^, ^28^ ^]^ It has been demonstrated to be a powerful tool to probe the magnetic susceptibility via the Evans method and extract the thermodynamic parameters of the transition and hence the spin‐state population via the evolution of certain chemical shifts over the SCO temperature range.^[^ ^26^, ^27^, ^28^, ^29^ ^]^ Furthermore, as several SCO complexes are only stabilized in solution,^[^ ^21^ ^]^ it has been utilized to study their transition as well as to monitor the potential solvent effects on both the complex stability and spin‐transition.^[^ ^30^ ^]^ In terms of transition metal centers, most works have focused on Fe(II) because the transition from the diamagnetic spin‐singlet state to the paramagnetic quintet state allows a relatively straightforward identification of the individual environments and tracking of the evolution of the individual paramagnetic shifts across the transition.^[^ ^20^, ^23^, ^26^, ^27^, ^31^ ^]^ Solution NMR studies of SCO complexes containing Fe(III),^[^ ^30^ ^]^ Mn(II),^[^ ^32^ ^]^ and Co(II)^[^ ^33^ ^]^ have been reported as well.

However, solution NMR is not suited to study solid SCO systems, which are of high interest for applications as memory devices and molecular switches.^[^ ^34^, ^35^, ^36^, ^37^ ^]^ These systems require a hysteretic memory effect that can only be found in the solid state. Solution NMR spectroscopy is therefore not suited to study these systems, even if dissolved, because solvent effects may alter spin‐state equilibria and lattice cooperativity is not accounted for. In addition, several SCO complexes undergo transitions at cryogenic temperatures, which is inaccessible for solution NMR spectroscopy. Another potential advantage of NMR measurements in the solid state is related to paramagnetic relaxation effects. Generally, these create a “blind sphere” near the transition metal,^[^ ^38^ ^]^ meaning that resonances in close proximity to the paramagnetic center become undetectable due to severe broadening effects. Several relaxation mechanisms contribute to this, but one is exclusive to solutions as it describes paramagnetic relaxation caused by molecular tumbling. This mechanism, the Curie relaxation, has been proven to be negated in spinning solids,^[^ ^39^ ^]^ potentially extending coherence lifetimes.

Solid‐state NMR of SCO systems has so far only been explored in a limited number of cases.^[^ ^22^, ^40^, ^41^ ^]^ Thus, a wider use of NMR for this purpose requires the development of a dedicated methodology that can cover SCO transitions over a wide range of temperatures, including low to ultra‐low temperatures. Herein we show that low temperature (LT) Magic Angle Spinning (MAS) solid‐state NMR can be used in combination with quantum chemistry calculations, to access site‐specific NMR shift information related to structural and electronic changes of a [MnL1]PF_6_ spin crossover complex during the SCO transition. More specifically, we show that ^13^C and ^1^H solid‐state MAS NMR methods can be employed from room temperature down to 100 K to acquire high‐resolution spectra. The prediction of the hyperfine parameters and the paramagnetic NMR (pNMR) shifts with quantum chemistry calculations allows assigning and tracking the evolution of specific ^1^H and ^13^C resonances in both spin‐states during the spin‐transition. Consequently, we have correlated the observed ^1^H/^13^C pNMR shifts of [MnL1]PF_6_ with ESD changes throughout the coordination ligands across a SCO transition. This work thus establishes that LT MAS NMR is an excellent tool for the atomic‐scale study of molecular magnetic systems with spin‐state transitions. It provides insight into both the subtle changes in electron density distribution in the ligands of SCO molecules and the macroscopic cooperative behavior during these transitions.

## Results and Discussion

### [MnL1]PF_6_: A Spin Crossover Complex with Low Temperature Transition

**Figure 1:**
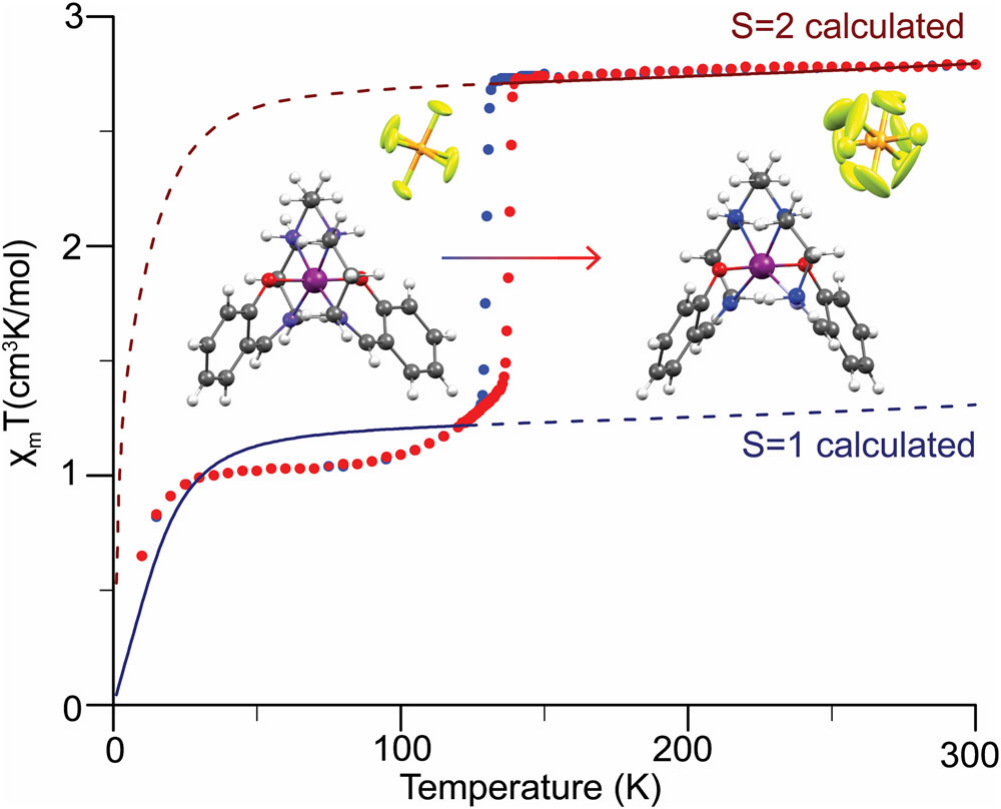
Experimental and calculated χ_m_ *T* versus *T* curves of [MnL1]PF_6_. The blue and red filled circles correspond to the experimental cooling and heating modes, respectively (adapted from Ref. 18). The dashed blue and red lines correspond to the calculated LS (*S* = 1) and HS (*S* = 2) χ_m_ *T* versus *T* curves. The insets illustrate the [MnL1]PF_6_ building block in the LS state (left) and the HS state (right). Purple color marks the Mn, orange the P, green the F, red the O, blue the N, dark gray the C, and white the H atoms, respectively.

The complex [MnL1]PF_6_ consists of a Mn^3+^ ion that is coordinated to Schiff base ligands forming the complex [Mnsal_2_(323)]^+^, sal_2_(323) = 2, 2´‐(2, 6, 9, 13‐tetraazatetradeca‐1, 13‐diene‐1, 14‐diyl)diphenolate = L1.^[^ ^18^ ^]^ The [MnL1]PF_6_ mononuclear complex was synthesized following the synthetic procedure established by a previous work^[^ ^18^ ^]^ (see Materials section in the Supporting Information) and its structure was verified via powder X‐ray diffraction at room temperature (Figure S1). This complex has a pseudo‐octahedral coordination around the Mn^3+^ ion with two axial phenolate groups and two pairs of *cis*‐imine and *cis*‐amine groups on the equatorial axis with mirror symmetry. These molecules are then aligned in a one‐dimensional manner, with PF_6_^−^ anions in between, acting as counterions. More specifically, below the SCO transition, [MnL1]PF_6_ is in the LS state (*S* = 1) and adopts an orthorhombic *P*2_1_2_1_2_1_ crystal structure.^[^ ^18^ ^]^ Upon heating above the SCO transition, the space group of the crystal structure is retained, yielding only a slight change in the lattice parameters, while the electron configuration changes from a *t*_2g_^4^ *e*_g_^*0^ configuration (LS, *S* = 1) to *t*_2g_^3^ *e*_g_^*1^ (HS, *S* = 2), which is associated with bond length changes between Mn and the ligands. In addition to a strong JT effect, the hydrogen bond between amine hydrogen of the ligand and the fluorine atoms of the PF_6_^−^ anion is also broken, leading to a loss of ordering for the PF_6_^−^ anions. The corresponding thermal SCO transition is clearly seen in the magnetic molar susceptibility (χ_m_) multiplied by the temperature, plotted as a χ_m_ *T* versus T curves (Figure 1), where (i) by decreasing temperature the HS state starts to turn into the LS state, at 133 K, transforming completely at 100 K, while (ii) upon heating the reverse transition starts to appear at 138 K, therefore forming a hysteresis loop as presented in Figure 1.

However, while the role of the charge balancing counterions is well understood and the crystal structures of the HS and LS states known, we still need an approach that can probe atomic‐scale structural information sensitive to changes in ESD distribution. Such information can provide important insights for refining coordination changes and for positioning light atoms, ultimately enabling the rational design of next‐generation spin‐crossover materials for high‐density data storage applications. To move toward this goal, we implemented variable low‐temperature solid‐state NMR spectroscopy, supported by ab initio calculations of the electronic properties of each spin state.

### Quantum Chemical Calculations

**Figure 2:**
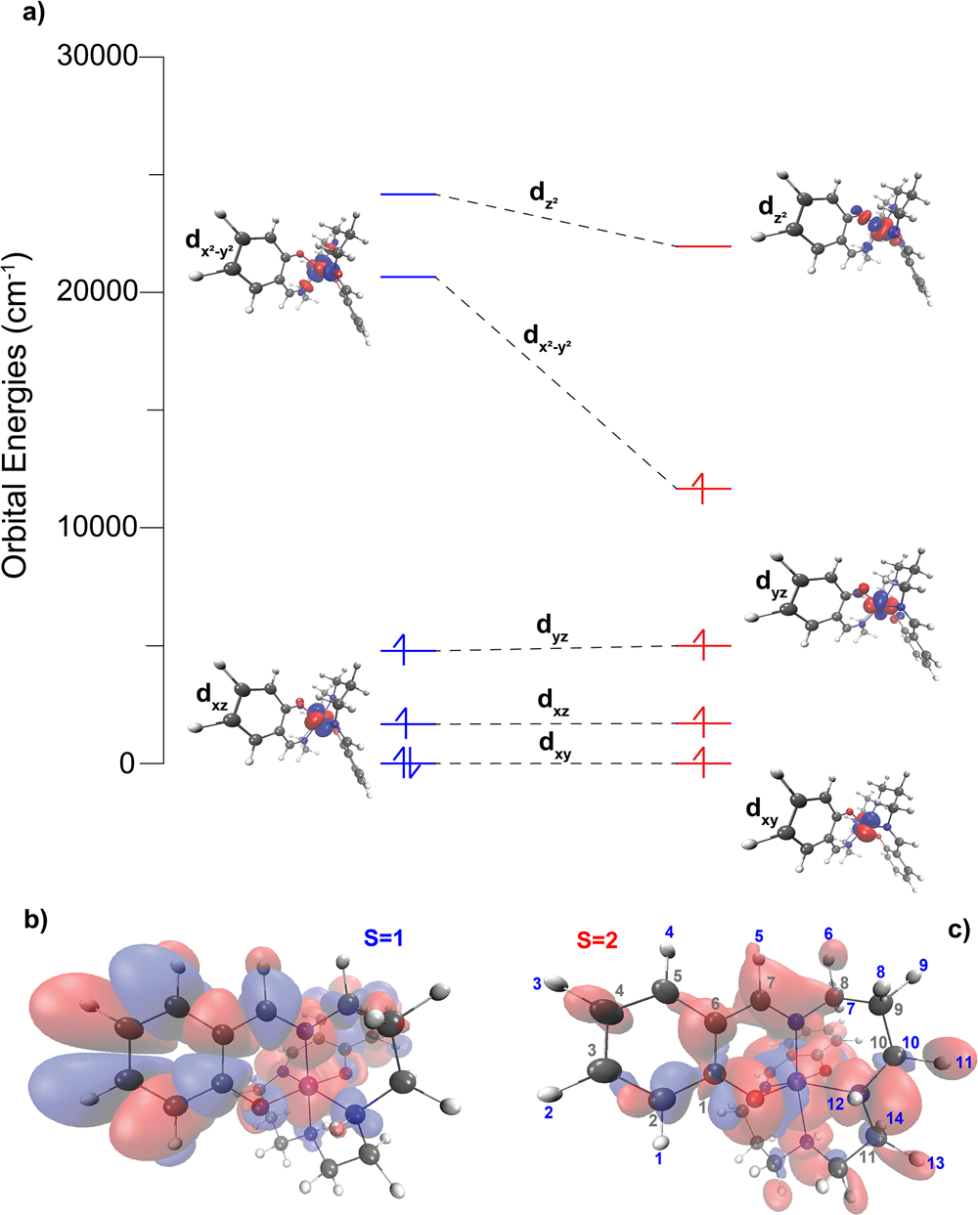
a) Ab initio ligand field theory (AILFT) analysis of the d orbital energies and b) and c) transferred electron spin densities of the two spin states of [MnL1]PF_6_. For (a), orbital energies in blue correspond to the LS state, while the energies in red correspond to the HS state. Upon the spin crossover, the orbital energies of both $d_{z^{2}}$ and especially $d_{x^{2}-y^{2}}$ become lower, while the d_*yz*_ orbital energy increases slightly. The former is a signature of equatorial elongation of the N_4_ donor set due to a pronounced JT effect. The red color corresponds to positive isosurface and blue color to negative isosurface with values of ± 0.04 a.u. (b) The pronounced transferred ESD onto the phenolate ring in the *S* = 1 state is shown. The isosurface was plotted with a value of ± 0.001 a.u. (c) In the *S* = 2 state, the ESD resides primarily on the N_4_ backbone. The isosurface value corresponds to ± 0.0001 a.u. The ESD calculations have been performed at CCSD‐DLPNO level of theory.

All calculations presented in this work were performed with the quantum chemical calculations package ORCA^[^ ^42^ ^]^ by applying the complete active space self‐consistent field (CASSCF)^[^ ^43^, ^44^ ^]^ method, combined with second order n‐electron valence state perturbation theory (NEVPT2)^[^ ^45^, ^46^, ^47^ ^]^ calculations. The initial HS and LS structures have been taken from Ref. 18 The very good agreement between the experimental and calculated χ_m_ *T* versus *T* curves for both spin‐states (Figure 1) corroborates the previous crystallographic and magnetic assignment of [MnL1]PF_6_.^[^ ^18^ ^]^ In addition, ab initio ligand field theory (AILFT)^[^ ^48^ ^]^ analysis was performed (Figure 2). It shows a considerable decrease in the energy of the $d_{x^{2}-y^{2}}$ orbital in the HS state, allowing its occupation. This is the signature of the strong equatorial JT elongation, reflected in the increase of the Mn─N_amine_ bond lengths in this state, as shown in Table 1.

**Table 1:** The bond lengths between the Mn(III)‐center and the coordinating N‐ and O‐atoms for both spin states, after DFT geometry optimization.

Bond	LS (Å)	HS (Å)
Mn─N_amine_	2.06	2.26
Mn─N_imine_	1.98	2.09
Mn─O_phen_	1.87	1.86

The observed NMR shifts can be broken down into δ_obs_ =  δ_para_ +  δ_dia_, with δ_dia_ corresponding to the diamagnetic orbital shift, while the temperature dependent δ_para_ arises from the interaction of the nuclear spin with the thermally averaged electron magnetic moment parameterized by the full EPR Hamiltonian, holding valuable information on the magnetic structure of the system. The paramagnetic shift term can be further broken down into δ_para_ =  δ_FC_ +  δ_PC_ +  δ_PSO_, with δ_FC_ being the scalar Fermi‐contact term, δ_PC_ the intra‐molecular pseudo‐contact (dipolar) term and δ_PSO_ the paramagnetic spin‐orbit coupling term. Specifically, δ_PC_ arises from the dipolar through‐space interaction between the average electron moment and the nucleus and can be significantly affected by the anisotropy of the ***g*** tensor.^[^ ^49^ ^]^ On the contrary, the Fermi‐contact shift stems from the delocalization of the unpaired electrons onto the nucleus that is being probed. Due to its through‐bond nature, the spectra directly reflect the amount and sign of transferred ESD from the Mn^3+^ ion onto the s‐orbitals of the ligand's local environments. In the case of paramagnetic systems, the computation of the NMR shifts and shift anisotropies thus require considering the full EPR Hamiltonian, including the diamagnetic orbital shielding tensor σ_orb_, the hyperfine coupling tensor *A* between the nuclear ($\hat{I})$ and electron ($\hat{S})$ spins, as well as the ZFS tensor *D* term^[^ ^49^ ^]^:

(1) $$\begin{array}{cc} {\hat{H}}_{\mathrm{EPR}} & =-\hbar \gamma B_{0}\cdot \left(1-\sigma_{\mathrm{orb}}\right)\cdot \hat{I}+\mu_{B}B_{0}\cdot g\cdot \hat{S}\\ & \;+\hat{S}\cdot A\cdot \hat{I}+\hat{S}\cdot D\cdot \hat{S} \end{array}$$

with ℏ being the reduced Planck constant, γ the gyromagnetic ratio, *B*_0_ the magnetic field, μ_**B**_ the Bohr magneton and *g* the free electron g‐tensor. Considering Equation 1, by employing quantum chemistry calculations with the aid of the ORCA package,^[^ ^42^ ^]^ it is possible to calculate *A* and *D*, and subsequently the total NMR shielding tensor which enables the experimental NMR peaks assignment. Within the modern implementation by Vaara et al. of the Kurland and McGarvey theory,^[^ ^49^, ^50^, ^51^, ^52^ ^]^ the total NMR shielding can be expressed in terms of the EPR property tensors as

(2) $$\sigma =\sigma_{\mathrm{orb}}-\frac{\mu_{B}}{\hbar \gamma \mathrm{kT}}g\cdot \langle \mathrm{SS}\rangle \cdot A$$

where 〈**SS**〉 is the dyadic tensor of the ZFS Hamiltonian. The ***g*** and ***D*** tensors were calculated with the CASSCF/NEVPT2 method using the relativistic second‐order Douglas–Kroll–Hess Hamiltonian, while *A* was calculated by using a CCSD‐DLPNO (coupled cluster domain based local pair natural orbital)^[^ ^53^, ^54^ ^]^ approach. The breakdown of each tensor and their calculation protocol are described in more detail in the Supporting Information. We note that δ_PSO_ is often negligible for 3d transition metals except in rare cases such as high‐spin Co^2+^. In addition, we also observe that inter‐molecular contributions to the pseudo‐contact term, which can be calculated using the semi‐empirical point‐dipole approach is shown to be negligible (Figure S4). The complete breakdown of the contributions of δ_FC_, δ_PC_, and δ_PSO_ to the total calculated pNMR shift at the most relevant experimental temperatures are given in Figures S5–S7 for ^1^H and Figures S8–S10 for ^13^C, respectively.

Finally, the isotropic NMR shift was referenced using the calculated absolute shielding of CH_4_ (σ_calc,ref, 1H _ = 31.643 ppm, σ_calc,ref, 13C _ = 192.044 ppm) and the experimental shielding in the CH_4_ gas phase^[^ ^55^ ^]^ (σ_exp,ref,  1H_ = 2.166 ppm, σ_exp,ref, 13C_ = −8.648 ppm) according to formula:

(3) $$\delta =\sigma_{\mathrm{calc},\mathrm{ref}\;}-\sigma_{\exp,\mathrm{ref}}-\sigma_{\mathrm{dia}}-\sigma_{\mathrm{para}}$$

### Solid‐State NMR: Assignment of the Paramagnetic Shifts at Room Temperature (*S* = 2)

**Figure 3:**
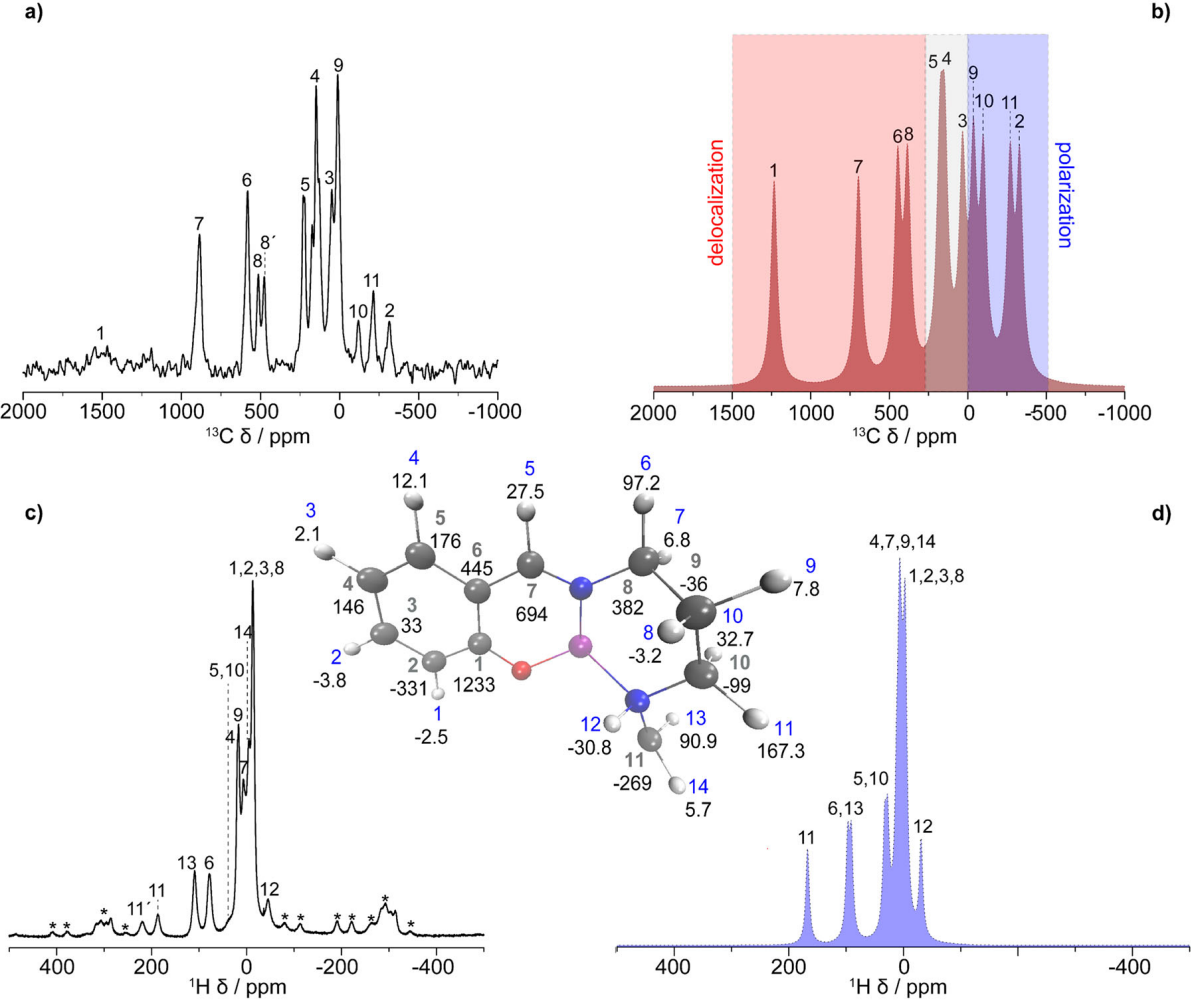
a) and b) The experimental (a) and calculated (b) ^13^C MAS NMR spectra of [MnL1]PF_6_ recorded at a magnetic field strength of 4.7 T and a MAS rate of 60 kHz. c) and d) The respective experimental (c) and calculated (d) ^1^H MAS NMR spectra. Asterisks denote spinning sidebands in the experimental ^1^H spectrum. Only the isotropic shifts were considered for the calculated spectra, which were simulated with the WSolids software.^[^ ^68^ ^]^ Inset: The label of each individual atom in [MnL1]PF_6_ (blue for H, gray for C) and its corresponding isotropic shift in ppm (black numbers). Only half of MnL1 is depicted, as the molecule has a rotational symmetry between the ligands. White corresponds to hydrogen atoms, gray to carbon, red to oxygen, blue to nitrogen and violet to the manganese atom.

The L1 ligand contains 14 unique hydrogen sites and 11 unique carbon sites (inset in Figure 3), which are duplicated by rotational symmetry in the [MnL1]PF_6_ complex. The corresponding ^13^C and ^1^H one‐dimensional very fast MAS solid‐state NMR spectra (ν_r_ = 60 kHz), recorded at ∼320 K at a magnetic field strength of 4.7 T are presented in Figure 3a,c. The low magnetic field and fast MAS was utilized to dampen the paramagnetic shift anisotropy, as well as the inhomogeneous broadening of the resonances due to bulk magnetic susceptibility (BMS) effects, that scale with the external field strength.^[^ ^56^, ^57^, ^58^ ^]^ The ^13^C spectrum recorded at 4.7 T (Figure 3a) is indeed devoid of visible spinning‐sidebands and contains 11 distinguishable shifts that span 1800 ppm. This includes a peak at approx. 1450 ppm that is extremely broadened compared to the other resonances. For ^1^H, the slight overlap of the isotropic shifts with spinning‐sidebands in the ^1^H spectrum (Figure 3c) hinders the detection of all ^1^H isotropic shifts. This can be resolved in the 2D projection Magic Angle Turning—Phase Assisted Sideband Separation (pj‐MATPASS) experiment^[^ ^59^ ^]^ (Figure S3), in which one additional peak at 221 ppm becomes visible.

It is interesting to note that the use of a higher magnetic field than 4.7 T (for a given MAS frequency) results in overlapping spinning‐sidebands and isotropic peaks,^[^ ^60^ ^]^ obstructing a precise detection of some of the resonances (Figure S2). Nevertheless, 1D spectra collected at 9.4 T can still be used for cross‐validation. In addition, the ^1^H resolution (for a given MAS frequency) is slightly improved for resonances with moderate hyperfine shifts (see insets of Figure S2b,d with resonances between −15 to 30 ppm). The corresponding calculations are shown in Figure 3b,d for ^13^C and ^1^H, respectively.

Remarkably, a satisfactory match between experimental and calculated shifts is obtained for the ^13^C shifts of MnL1 in the high‐spin state (*S* = 2), allowing complete resonance assignment (inset of Figure 3). It is interesting to note that, for the HS case (at 320 K), the Fermi‐contact contribution is dominant (see Figure S8 for the relative contributions of the FC, PC, and PSO contributions), which explains why atoms in close through‐bond proximity to the Mn^3+^ ion (e.g., **C1**, **C6**, **C7**, and **C8**) are the most shifted. This also translates into a large transferred ESD, as shown in Figure 2c.

Starting from the phenolate group (C1–C6), **C1** is the most shifted carbon at 1450 ppm, with the next nearest being **C2** and **C6** at −314 and 581 ppm, respectively. We note that these values are consistent with a previous report by Levin et al. on Mn(acac)_3_.^[^ ^61^ ^]^

The negative shift of the **C2** (−314 ppm) is attributed to spin‐polarization, a mechanism that involves a negative transferred ESD. The excess of α‐spin density on the atoms contributing to the SOMO draws more α‐electron spin density from its neighbor leaving an excess of β‐electron spin density, hence polarization. Concerning the **C3**, **C4**, and **C5** carbons, the corresponding shifts are smaller because these carbons are more distant from the Mn^3+^ ion and in consequence possess the least transferred spin density.

Moving on to the N_4_ donors, the negative shifts observed in **C11** and **C10**, which are directly bonded to the N_amine_, are due to spin‐polarization. The elongation of the Mn─N_amine_ leads to the N_amine_ drawing positive ESD, causing significant negative ESD on its neighboring atoms. Possible electron delocalization and polarization mechanisms between transition metal centers and surrounding atoms have been extensively described elsewhere.^[^ ^62^, ^63^, ^64^ ^]^ A small extent of spin‐polarization is observed also for **C9**, in accordance with the bond‐distance argument with respect to the paramagnetic center. For the **C8** carbon, which is part of the imine group, it is interesting to note that two peaks are observed in the experimental spectrum compared to only one in the calculated spectrum. This can be explained by the fact the geometry‐optimized structure, used for the calculations, is almost symmetrical. The **C7 carbon**, directly linked to the N_imine_ has the strongest positive shift (885 ppm) of the carbon resonance from the N_4_ donors.

In the case of ^1^H resonances, the comparison between the experimental and calculated MAS spectra, shown in Figure 3c,d, only allows a partial assignment of the ^1^H shifts. The contributions from the FC, PC, and PSO terms are given in Figure S5. The resonances associated with the highest shifts **H6**, **H11**, **H12**, and **H13** are clearly dominated by the FC term. The pronounced upfield shift of **H12** (−35 to −45 ppm) is attributed to its bonding with the nitrogen atom. In a similar manner to the amine carbon atoms, the significant α‐electron population of the N_amine_ atom mandates the population of β‐electrons of the σ‐orbital of **H12**, resulting in negative ESD that induces electron polarization. In the case of the **H6**, **H11**, and **H13** methylene protons, the transferred ESD is considerable, compared to their **H7**, **H10**, and **H14** counterparts bonded to the same respective carbon, which do exhibit much smaller paramagnetic shifts (see Figure 3). This can be explained through a hyperconjugation effect^[^ ^65^, ^66^, ^67^ ^]^ that is mediated through electron delocalization of the ligand σ orbital framework via Mn─N bonds for both amine and imine ligands that couples the nitrogen lone pairs electronically with primarily the **H6**, **H11**, and **H13** sites. This is reflected by the ESD distribution in Figure 2c. The other shifts present in the spectrum spanning from −15 to 30 ppm can only be assigned tentatively, due to the narrow experimental and calculated shift range.

### Probing both Spin‐States Through ^13^C LTMAS NMR

With the SCO transition taking place at the spin‐transition temperature (*T*_ST_) of ∼133 K upon cooling, as shown in the magnetic susceptibility measurements in Figure 1, we resorted to low temperature MAS (LTMAS) NMR experiments to probe the LS state below the SCO transition (at 100 K) and the HS state above the transition (at 175 K).

Acquiring and interpreting LTMAS NMR data is significantly more challenging than at room temperature due to several key factors. First, there is a limited availability of NMR probes capable of operating at fast MAS and moderate magnetic fields under cryogenic conditions. Second, low temperature amplifies hyperfine interaction effects, as both paramagnetic shifts and shift anisotropies vary inversely with temperature. This results in large isotropic shifts, often exceeding ±1000 ppm, which can challenge effective pulse excitation and leads to significant spectral broadening at a given MAS frequency. In addition, these broadened signals often overlap with spinning‐sidebands, making them difficult to resolve without the use of two‐dimensional NMR techniques. Additionally, the frictional heating caused by fast MAS rotation can elevate the sample temperature inside the rotor, which may deviate from the externally measured temperature. This is critical for monitoring the spin‐transition as it occurs in a narrow temperature window of 7–8 K.

**Figure 4:**
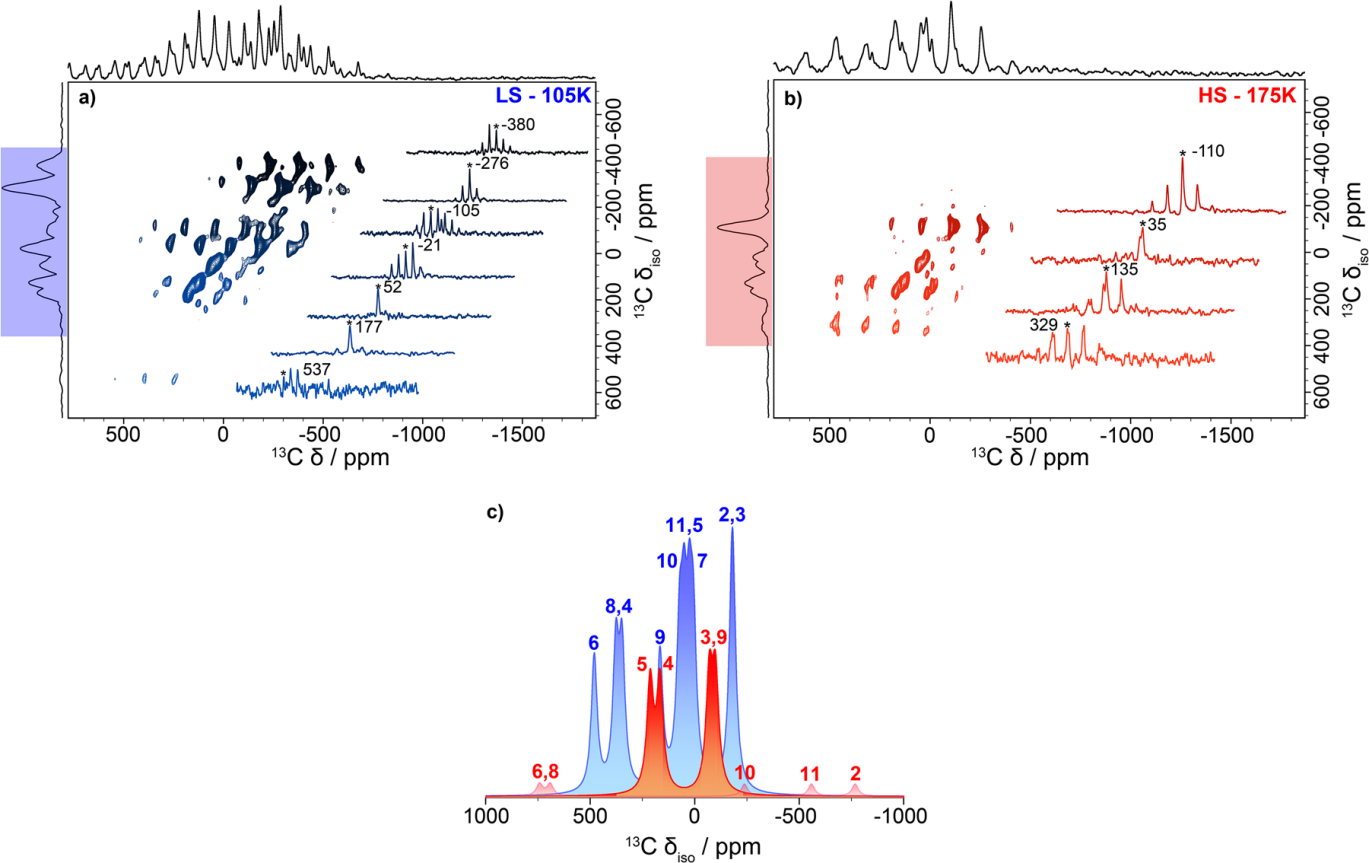
Detection of the LS and HS states in the heating mode with ^13^C aMAT NMR. a) and b) 2D ^13^C aMAT NMR spectra at 30 kHz spinning frequency in magnetic field of 18.8 T. The left panel (4a in blue color) is at 105 K (LS state), and the right panel (4b in red color) at 175 K (HS state). The 1D insets in both spectra are cross sections at the indicated *δ*_iso_, illustrating the spinning‐sideband manifold of each resonance. On the top and left sides of the spectra are the spectral projections. The panel in the middle below c) corresponds to the calculated isotropic shifts at temperature 105 K (LS‐blue color) and 175 K (HS‐red color). The calculated isotropic spectra were simulated with WSolids and the resonances not detected in the aMAT spectrum at 175 K (HS) were attenuated for clarity.

These challenges are well illustrated in Figure S11 that shows 1D ^13^C spectra at 105 K (LS) and 175 K recorded at 18.8 T with 30 kHz MAS using a rotor‐synchronized double‐adiabatic echo pulse sequence.^[^ ^69^ ^]^ We note that the higher magnetic fields and moderate spinning frequencies (compared to the spectra shown in Figure 3) can clearly explain the presence of strong spinning‐sidebands that makes the identification of isotropic shifts challenging. Nonetheless, the MAS frequency is high enough to allow implementation of the ^13^C and ^1^H 2D adiabatic magic‐angle turning (aMAT) pulse sequence (Figure 4).^[^ ^70^ ^]^ Such experiments correlate the isotropic NMR spectrum in the indirect dimension to the full spectrum (including spinning‐sidebands) in the direct dimension. Experimental details are provided in the Supporting Information.

The corresponding ^13^C aMAT spectra at 18.8 T and 30 kHz MAS are shown in Figure 4a,b for the LS (105 K) and the HS (175 K) states, respectively. We observe fewer resonances in the 175 K ^13^C aMAT HS spectrum recorded at 18.8 T and 30 kHz (indirect dimension of Figure 4b) compared to the 320 K ^13^C HS recorded at 4.7 T and 60 kHz (Figure 3a). This is the result of the larger shift dispersion and shift anisotropy at lower temperature, combined with the signal losses during the long rotor‐synchronized aMAT pulse sequence at 30 kHz MAS. Consequently, resonances with too short transverse relaxation times^[^ ^71^ ^]^ and/or chemical shifts outside the spectral excitation bandwidth are absent from the HS aMAT spectrum (175 K), which comprises only four groups of observable peaks. The same argument also applies to the 105 K ^13^C aMAT LS spectrum (indirect dimension of Figure 4a) but the spin state change translates into a reduction in paramagnetic shift and shift anisotropy, which allows the detection of most of the ^13^C resonances. The full list of hyperfine couplings of the carbon atoms in both the HS and LS states is provided in Table S2.

The comparison of the calculated shifts (Figure 4c) and the indirect dimension of the aMAT spectrum (which corresponds to an isotropic spectrum *δ*_iso_) is instrumental for the resonance assignment. For the HS spectrum at 175 K, the more intense ^13^C NMR peaks correspond, in descending frequency order, to carbon atoms **C5**, **C4**, **C3**, and **C9**, while the low intensity peaks correspond to strongly shifted resonances (positive and negative), attenuated by polarization losses during the pulse sequence. In the LS spectrum at 105 K, the calculated frequencies span a frequency range of ∼750 ppm (besides **C1**, which has a calculated isotropic shift of ∼2404 ppm). A slight discrepancy between experimental and calculated frequencies can be noted. This can possibly be attributed to the high value computed for both (i) the *g*‐factor (*g*_iso,calc_ =  2.16 compared to *g*_iso,exp_ =  2.08^[^ ^18^ ^]^) and (ii) the zero‐field‐splitting (*D*  =  40 cm^−1^ compared to 20 − 25 cm^−1^ typically found for spin‐triplet forms of related Mn(III)‐Schiff base complexes^[^ ^15^ ^]^). In addition, it is important to stress that the current limitation of this methodology also lies in the limited accuracy of the computation of the *A*−tensor. Proposals to improve on these results include conducting DFT calculations with hybrid functionals and varying Fock exchange parameters. While potentially more accurate, no definitive theoretical basis exists for the optimal Fock exchange percentage.^[^ ^72^ ^]^ Therefore, CCSD calculations which can account for electron correlation were performed in this work.

To understand the origin of the NMR shifts, we need once more to look into the ESDs across the MnL1 molecule in the LS and HS state (Figure 2b,c). In the LS state, considerable ESD resides on the phenolate carbons, absent in the HS state, which accounts for the higher “spread” in the ^13^C NMR shifts of specific phenolate carbons in the LS state in comparison to the HS state, such as **C3**, **C4**, and **C5**. The evolution of alternating electron delocalization and polarization on the carbons of the phenolate ring through the delocalized π‐space is explained through perturbation theory.^[^ ^64^, ^67^, ^73^ ^]^ Meanwhile, the carbon resonances on the N_4_ backbone are less affected by the through‐bond transferred ESD, with the pseudocontact term contributing equally to their paramagnetic shifts. In the HS state, as mentioned earlier, most of the ESD localizes on the imine and amine carbons, inducing strong paramagnetic shifts and reduction of transverse relaxation times of those sites. This limits the detection of resonances to sites that are less affected by the delocalized electrons.

Overall, the ^13^C aMAT NMR experiment provides excellent quality data that can be used to investigate the LS and HS Mn(III) electron spin states. Nevertheless, the approach is limited by the low receptivity of the ^13^C nucleus and requires a large number of transients per increment, which translates into long experimental time (∼14 h) to obtain high quality data. This clearly limits our ability to track changes in ESD distribution on the MnL1 complex through the spin‐crossover transition. As an alternative, we evaluate in the following whether ^1^H LTMAS NMR can be used toward this end.

### Tracking the Spin‐Crossover Hysteresis Loop Using ^1^H Variable Temperature LTMAS NMR Spectroscopy

In the previous section, limitations were highlighted in terms of information that can be extracted through the one‐dimensional ^13^C ssNMR spectra (Figure S11). These also apply to the proton shifts at low temperatures (Figure S12). The spectra, spanning approximately from −400 to +400 ppm, primarily consist of overlapping spinning‐sidebands from multiple ^1^H resonances. Despite the complexity of the dataset and challenges in assigning specific resonances, the spin‐crossover transition around 133–140 K is clearly observable through changes to the spinning sideband manifold and a clear drop in ^1^H signal intensity during the LS‐to‐HS transition (Figure S12). To address these challenges, the aMAT sequence was again employed, starting at 102 K with multiple measurements up to 175 K to ensure full conversion into the *S* = 2 state. At 102 K (LS), the aMAT spectrum is composed of three distinct groups of shifts, while four are observed for the HS state at 175 K (Figure S13). For both states, most of the calculated shifts align with the experimental data range, as shown in the overlap of the experimental projections and calculated shifts (Figure S13c).

We also note that the narrower ^1^H shift range and the limited resolution in the indirect dimension of the aMAT prevent resolving most of the ^1^H resonances. This limitation primarily arises from the limited evolution time that could be used in the indirect dimension of the aMAT experiments (only two rotor periods here). Yet, this is a mandatory compromise as the strong transverse relaxation present throughout the molecule would otherwise lead to coherence decay during the pulse sequence duration at 30 kHz MAS.

**Figure 5:**
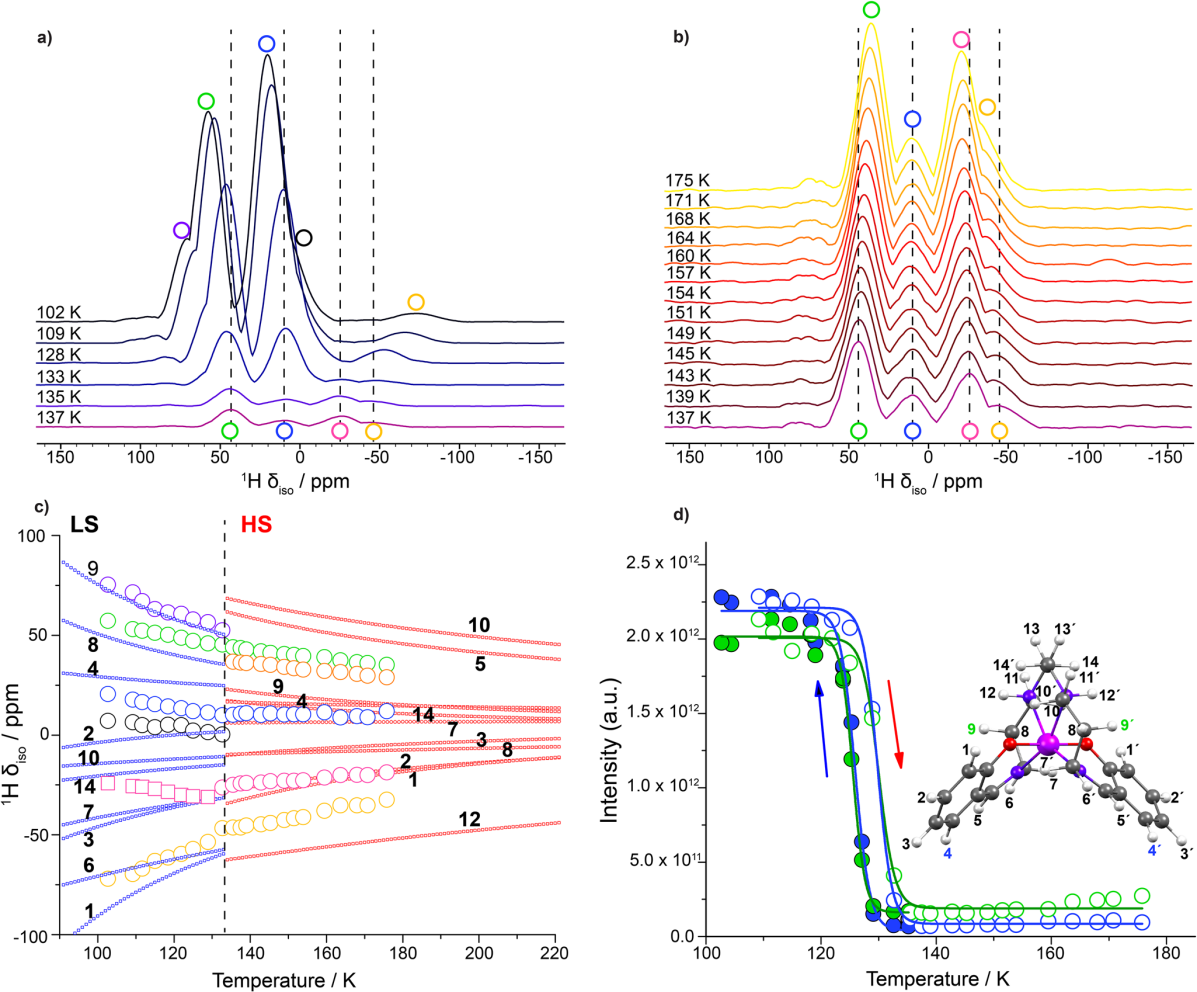
a) and b) The isotropic projections of the ^1^H aMAT NMR spectra acquired upon heating in magnetic field of 18.8 T, and at temperatures between 102 and 175 K. A notable decrease of the NMR signal intensity is observed as *T*→*T*_ST_. For better clarity, the intensities in Figure 5b, which contain the projections at temperatures in the HS regime, have been rescaled with respect to the LS spectra. c) The temperature dependence of the isotropic ^1^H NMR frequencies of all identified peaks (open circles), obtained by multi‐Gaussian fits of the isotropic projections, as illustrated in Figure S14 for an aMAT projection in the HS state. Pink open squares belong to ^1^H VT MAT NMR measurements at 9.4 T, by fitting the relevant isotropic projections. The dotted circles correspond to the ^1^H shifts determined from the aMAT traces at the respective frequency. The dotted lines are the calculated shifts of hydrogens, as numbered in the inset of panel 5d. d) The temperature dependence of the ^1^H NMR signal intensity *I* of the two more intense peaks of the LS state (green and blue circles in panel 5c). Hollow circles correspond to the heating mode, while full circles correspond to the intensity of the peaks while cooling down. These peaks are attributed to hydrogen atoms **H9** and **H4**. A hysteresis loop is observed upon heating/cooling, in agreement with the χ_m_ *T* versus *T* curve in Figure 1.

The temperature dependence of the isotropic ^1^H NMR spectra, obtained by projecting 2D aMAT spectra, is showcased in Figure 5a,b during heating. Given the limited resolution, it is difficult to track individual resonances and their evolution throughout the transition. The deconvolution of the isotropic LS spectra (indirect dimension of the aMAT spectra), using multi‐Gaussian fits combined with the evaluation isotropic shifts falling on the diagonal of 2‐D the aMAT spectra (Figure S13a) reveals four main shifts in the 0–100 ppm range that we tentatively assign to **H2**, **H4**, **H8**, and **H9**, in increasing chemical shift, based on the calculations. The resonances evolve progressively toward smaller absolute shifts with increasing temperature as shown in Figure 5c. During the spin transition, the redistribution of electron spin density (ESD) leads to abrupt changes in the magnitude and sign of proton resonances, particularly for environments closer to the Mn^3+^ center. Additionally, a marked drop in the overall signal intensity is observed at the onset of the transition at ∼130 K as $T\to T_{\mathrm{ST}}$ (Figure 5a).

We note that this relative signal loss is more pronounced in the aMAT spectra as compared to one‐dimensional spectra. While adiabatic pulses improve the excitation bandwidth in MAT experiments, their longer duration can therefore lead to increased coherence losses. In that regard, isotropic shift–sideband‐separation pulse sequences that do not utilize adiabatic pulses and are of shorter duration such as the slightly less broadband pj‐MATPASS sequence or the much less broadband MAT sequence, can in principle alleviate part of those coherence losses and detect additional resonances. This is further illustrated in Figure S15, which shows 2D ^1^H MAT spectra in both spin‐states at 9.4 T and a spinning frequency of ν_r_ =  10 kHz. MAT is the predecessor pulse sequence^[^ ^74^ ^]^ of aMAT, with conventional rectangular pulses instead of the adiabatic ones. Notably, an additional signal at around −25 ppm is clearly observed (Figures S15a and S16), corroborating the resonances calculated in this frequency range.

The almost stepwise decrease of the signal intensity at *T*_ST_ (Figure 5d) is reminiscent of the temperature dependence of the order parameter in the LS phase. Since the signal intensity in the LS state is much larger than in the HS state, one can consider that the signal intensity *I* is roughly proportional to *N*_LS_. It can therefore be defined as I∼1−γ, where $\gamma =\frac{N_{\mathrm{HS}}}{N_{\mathrm{HS}}+N_{\mathrm{LS}}}$ is the fraction of molecules in the HS state, or alternatively in terms of the order parameter *q* as $I∼\frac{\left(1-q\right)}{2}$. By measuring *I* in both the cooling and the heating modes, the same hysteresis loop is observed as in the χ_m_ *T* versus *T* curves (Figure 5d). The spin transition range is not affected by the magnetic field strength as the same behavior is observed at 9.4 T as well (Figure S17).

The apparent decrease of the ^1^H NMR signal intensity as the temperature approaches and exceeds *T*_ST_ can possibly be explained by the following effects: (i) the longitudinal and transverse relaxation rates *R*_1_ and *R*_2_ are proportional to *S* (*S* + 1) so three times larger in the HS case. A larger *R*_2_ could lead to an overall drop in intensity and broadening of the ^1^H NMR signals. (ii) In the HS state, dynamic JT modulations (breathing modes of the JT distortions) are also expected to dynamically modulate the ESD and thus influence *R*_2_. For example, in the case of the methylene **H8**, **H9** hydrogens, dynamic JT distortions (i.e., changing of bond lengths and angles in the Mn–O–N octahedra) induce ESD fluctuations, via a dynamic hyperconjugation effect; the unpaired electron spin density on the hydrogens depends on the solid angle between **H8**, **H9** and the **C8**, **C9**, and **C10** plane. A similar reasoning holds for **H2** and **H4**.

## Conclusion

In this work, ^13^C and ^1^H solid‐state NMR methods in combination with quantum chemistry calculations of the NMR shifts have been employed to acquire information on the electronic structure and the ESD distribution of the [MnL1]PF_6_ SCO system in both the LS (*S* = 1) and the HS (*S* = 2) states. Both experiments and calculations indicate that the ESD in the HS state is axially distributed, mainly through the phenolate oxygens and imine/amine nitrogen atoms around the Mn ion. In the case of the LS state, on the other hand, the ESD spreads rigorously up to the benzoic rings. Evidently, the restriction of the ESD in the HS state is related to the JT‐induced increase of the equatorial bond lengths. At the same time, a remarkable decrease of the ^1^H NMR signal intensity characterizes the transition from the LS to the HS state and is coupled with the order parameter of the LS‐HS phase transition. This effect is markedly seen on two pairs of hydrogens, the methylene H8, and H9 and the aromatic H2 and H4, and it can be explained by an increase of transverse relaxation rate *R*_2_ in the HS state. Since the ESD on the benzoic rings is ultimately reduced in the HS state, it is anticipated that the *R*_2_ increase is merely of magneto‐vibronic nature, and it is eventually associated with the reported HS symmetric O‐Mn‐O and ring‐stretching modes, inducing ESD fluctuations via a hyperconjugation mechanism. The use of variable low‐temperature MAS NMR methods, coupled with ab initio calculations of the paramagnetic shifts, enabled us to resolve the electronic environment of certain ligand carbons and hydrogens of this particular Mn^3+^ mononuclear SCO system across the transition from the *S* = 1 to the *S* = 2 spin state. Ongoing improvements in fast MAS NMR hardware at low and ultra‐low temperatures should allow improving the quality of the data in the near future. The proposed low‐temperature ssNMR methodology is potentially an outstanding tool in the study of complex electronic phase transitions for SCO systems as it acts as a bridge between magnetic measurements and structural chemistry. By probing both the electronic state and the crystal structure in detail, it offers an additional layer of depth in the exploration of spin‐crossover phenomena and ultimately the design of functional molecular spin switches.

## Supporting Information

The authors have cited additional references within the Supporting Information.^[^ ^75^, ^76^, ^77^, ^78^, ^79^, ^80^, ^81^, ^82^, ^83^, ^84^, ^85^, ^86^, ^87^, ^88^, ^89^, ^90^, ^91^, ^92^, ^93^, ^94^, ^95^, ^96^, ^97^, ^98^, ^99^, ^100^, ^101^, ^102^, ^103^, ^104^, ^105^, ^106^, ^107^, ^108^, ^109^, ^110^, ^111^, ^112^, ^113^, ^114^, ^115^ ^]^

## Conflict of Interests

The authors declare no conflict of interest.

## Supporting information



Supporting Information

## Data Availability

The data that support the findings of this study are available from the corresponding author upon reasonable request.
