# Supplementary material for: Tracking Structural and Electron Spin Density Changes in a Cooperative Mn3+ Spin Crossover Complex at Atomic Scale via Low Temperature Solid‐State NMR
Source: Angew Chem Int Ed Engl. 2025 Dec 10;65(3):e17466. doi: 10.1002/anie.202517466 (PMC12811660; doi:10.1002/anie.202517466)
Supplement: Supplementary file 1 — Supporting Information [file ANIE-65-e17466-s001.docx]

**Tracking Structural and Electron Spin Density Changes in a cooperative Mn^3+^ Spin Crossover Complex at Atomic Scale via Low Temperature Solid-State NMR**

Wassilios Papawassiliou^[a]^, José P. Carvalho^[b]^, Subhradip Paul^[a]^, Aizuddin Sultan^[c]^, Michael Fardis^[d]^, Georgios Papavassiliou^[d]^, Grace G. Morgan^[c]^*, Katharina Märker^[a]^ and Gaël De Paëpe^[a]^*

**Affiliations**

^[a]^ Univ. Grenoble Alpes, CEA, IRIG-MEM, Grenoble, France,

^[b]^ Interdisciplinary Nanoscience Center (iNANO) and Department of Chemistry
 Aarhus UniversityGustav Wieds Vej 14, DK-8000 Aarhus C, Denmark,

^[c]^ School of Chemistry, University College Dublin, Belfield, Dublin 4, Ireland,

^[d]^ Institute of Nanoscience and Nanotechnology, National Center for Scientific Research “Demokritos”, 153 41 Aghia Paraskevi, Attiki, Greece

*grace.morgan@ucd.ie , gael.depaepe@cea.fr

Table of Contents

Synthesis and sample characterization.................................................................... S2

ssNMR spectroscopy.................................................................................................S3

Quantum Chemistry Calculations............................................................................. S4

Kurland McGarvey Formalism.................................................................................. S5

Results and Discussion............................................................................................ S6

References..............................................................................................................S15

**Synthesis and sample characterization**

[MnL_1_]PF_6_ was prepared according to the procedure previously reported ^[1]^. The Powder x-ray diffraction pattern was acquired at room temperature using an X-ray diffractometer (Malvern Panalytical B.V., Empyrean) operated at 40 kV and 30 mA of CuKα radiation (λ = 1.5406 Å) in 5–100º 2θ range with a step size of 0.03º. The pattern obtained is in full agreement with the previous crystal analysis report, as shown in Figure S1.

**
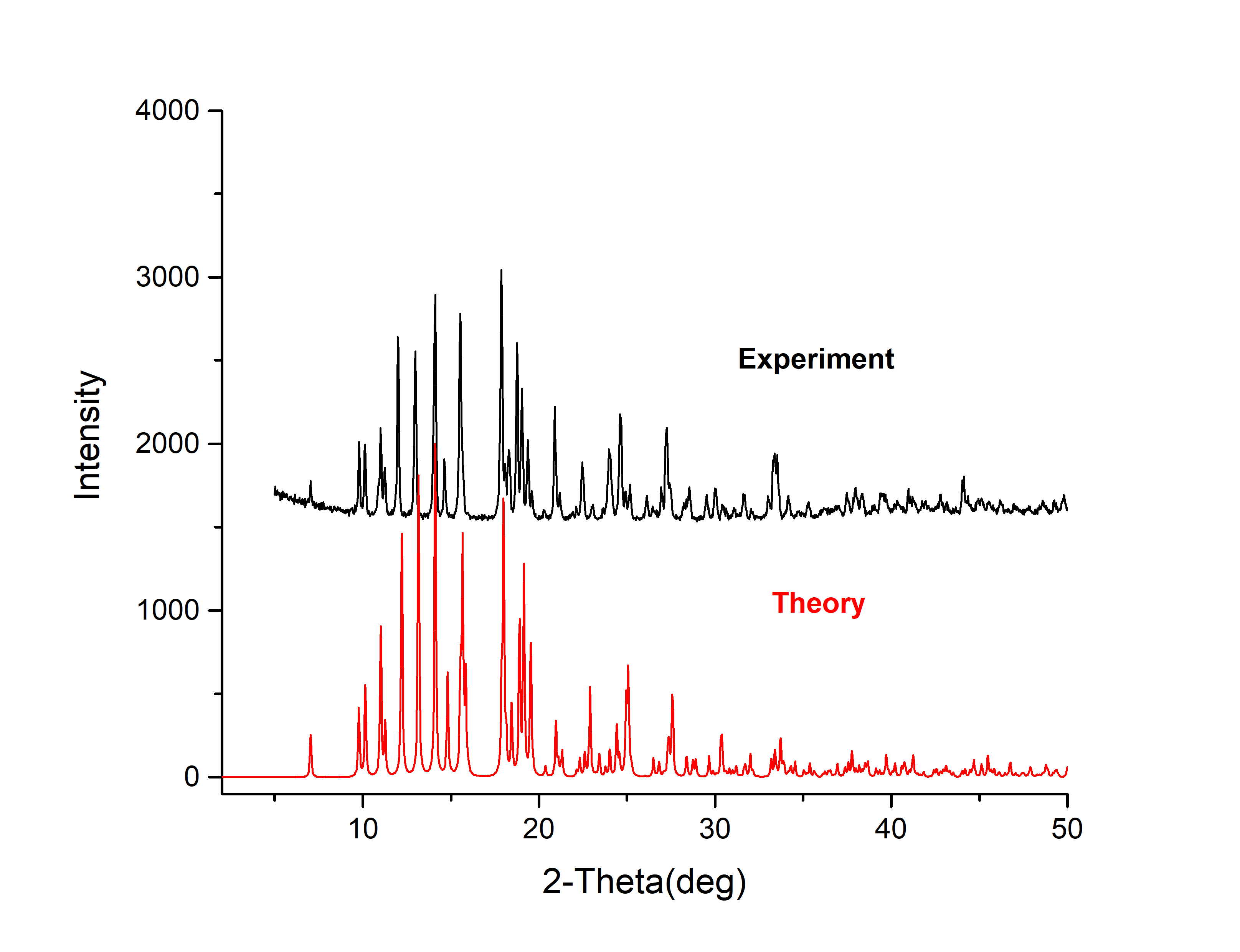
**

**Figure S1.** The experimental (black) and simulated (red) XRD powder patterns of **1**. The experimental powder pattern was acquired at room temperature in the High-Spin state, while for the simulated pattern the CIF file obtained at 142K from ref [1] was used (Code name in the Cambridge Crystallographic Data Centre CCDC-898551). The good match between experiment and simulation verifies the quality of the sample.

**ssNMR spectroscopy**

**Solid-State MAS NMR**

**The ^1^H and ^13^C MAS and variable LTMAS NMR experiments on the compound MnL1^+^ [PF_6_]^-^ were performed at three magnetic field strengths on three different spectrometers.**

**(i) Experiments at 4.7 T and Fast MAS.** The ^1^H and ^13^C MAS NMR spectra were recorded with a 1.3 mm HX double resonance probe, on a Bruker Avance-I spectrometer operating at Larmor frequencies of 200 MHz and 50.306 MHz for ^1^H and ^13^C, respectively. A rotor-synchronized Hahn-echo pulse sequence was used for acquisition with excitation pulse lengths of 2.5 μs and 3 μs corresponding to radiofrequency (RF) fields of 100 and 83.33 kHz for ^1^H and ^13^C. The same pulse length was used for recording the projection MATPASS spectrum. The repetition time was set to 50ms for ^1^H and 25ms for ^13^C. For the 1-D Hahn-echo spectra, 256 (^1^H) and 1433600 (^13^C) scans were accumulated.

**(ii) Experiments at 9.4T. (a) Fast MAS:** The ^1^H and ^13^C MAS spectra were collected with a 1.3 mm HXY triple-resonance probe, at 60 kHz MAS on a Bruker 400 Avance-III spectrometer operating at Larmor frequencies of 400.1 and 100.603 MHz for ^1^H and ^13^C respectively. The one-dimensional spectral acquisition was done with a double adiabatic spin-echo sequence (DAE) with a 1.5 μs 90° excitation pulse length, proportional to an RF field of 156 kHz, followed by a pair of rotor-synchronized short high-power adiabatic pulses (SHAPs) ^[2,3]^ of 50 μs length and a 5 MHz frequency sweep. The repetition time for all experiments was set to 10 ms. 5242880 and 1024 transients were accumulated for ^13^C and ^1^H, respectively. **(b) Variable Temperature LT MAS NMR:** Variable low-temperature MAT MAS NMR spectra were recorded on a LTMAS DNP 3.2mm HXY probe at spinning speeds of 10kHz on the same spectrometer. The excitation pulse length (90°) was set to 2.5μs corresponding to 100kHz RF field strength and the recycle delay was set to 50 ms. 84 signal transients per increment over 96 increments were accumulated for each experiment. The MAT experiment, like the aMAT experiment, has one excitation pulse followed by five refocussing π-pulses ^[4]^ (note: as SHAPs are pairwise, the aMAT sequence has 6 adiabatic pulses following the initial excitation pulse) the same RF field and recycle delay was used, with an evolution time of 100μs, equivalent to 1 rotor period. We note that we avoided the use of rotor synchronized SHAPs as (i) the adiabatic condition for complete magnetization inversion would not be fully met such low spinning frequencies and (ii) the additional rotor synchronization of those pulses would increase the duration of the sequence by a considerable amount of time (100us for each adiabatic pulse x6). This would eventually lead to the decay of all coherences.

**(iii) Experiments at 18.8T, Fast MAS and Variable Temperature LT MAS NMR:** The variable low-temperature MAS NMR spectra of MnL1^+^[PF6]^-^ were acquired with a LTMAS DNP 1.3 mm HXY probe, at spinning speeds of 30 kHz MAS, on a MAS on a Bruker 800 Avance Neo spectrometer operating at a ^1^H Larmor frequency of 797.7 MHz and a ^13^C Larmor frequency of 200.1 MHz, respectively. For the acquisition of the ^1^H DAE spectrum, an initial excitation pulse with length 2.125 μs corresponding to RF field amplitude of 117 kHz was used, and the following rotor synchronized SHAPs were sweeping through 5 MHz in 33.33 μs. 4096 signal transients were accumulated for each experiment. For the aMAT spectra, the same SHAPs were used with an evolution time of 66.66 μs, excluding the length of the SHAPs, which is equivalent to two rotor periods. 96 transients per increment for 128 increments were accumulated for each experiment. For all ^1^H experiments a recycle delay of 25 ms was used. For the ^13^C spectra, an initial excitation pulse of 3.25 μs was used corresponding to 76.9 kHz was used followed by the same scheme as for ^1^H. A repetition time of 10ms was used in the case of ^13^C. In all cases, chemical shifts were referenced to adamantane*.* The sample temperature has been determined from the spin-lattice relaxation time ($T_{1}$) of ^79^Br in KBr [5], which was measured at variable temperatures matching the temperature range of the experiments for the complex.

**Quantum Chemical Calculations**

All quantum chemistry calculations were performed using the program ORCA 5.0.1^[6]^ with an increased accuracy of numerical integration (orca keyword **defgrid3**, in particular with a strongly increased radial integration accuracy for Mn atom (orca keyword **SpecialGridIntAcc 12**) and very tight SCF convergence criteria, (orca keyword **verytightscf**). The resolution of the identity combined with the chain of spheres approximation were used (orca keyword **RIJCOSX**). The geometry optimizations of the [MnL1]^+^ molecular clusters were performed with **PBE0** functional ^[7-9]^, combined with the charge dependent atom-pairwise dispersion correction (**D4**) ^[10]^ for both high-spin and low-spin electronic configurations. The Weigend-Ahlrichs ^[11]^ **def2-TZVP** basis-set was employed for all atoms along with the def2-JK fitting basis-set was used for Coulomb and exchange integrals ^[12]^. The initial structure was taken from the crystallographic structures reported in Martinho et. al. ^[1]^. Tight geometry optimization criteria were considered (orca keyword **tightopt**). To ensure that the optimized structures correspond to local minimum of the potential energy surface, numerical frequencies calculation were also carried out to ensure the optimized geometry. No vibrational modes with imaginary frequencies were obtained. The calculation of the g and zero-field splitting tensors, magnetization and magnetic susceptibility was carried out considering a state-averaged, complete active space self-consistent field (**CASSCF**) wave function, distributing 4 electrons over five 3d orbitals of Mn, CAS(**4,5**) ^[13-15]^ using 5 quintet and 45 triplet equally weighted roots, followed by N-electron valence second-order perturbation theory (**NEVPT2**) ^[16-18]^ with the resolution of identity approximation, including both scalar relativistic second order Douglas-Kroll-Hess (**DKH**) Hamiltonian ^[19-21]^, as well as spin-orbit coupling using quasi-degenerate perturbation theory (**QDPT**) ^[22,23]^ and considering the spin-orbit mean-field approximation (**SOMF**) to compute the necessary the spin-orbit matrix elements ^[24,25]^, which included one-electron terms, Coulomb terms using resolution of identity approximation, exchange via one-center exact integrals including the spin-other-orbit interaction. Only the spin-orbit contribution to the zero-field splitting tensor was considered, since the spin-spin contribution is expected to be small ^[26]^. Both a finite nucleus model and second-order picture change effects were considered (using second order DKH transformation of the spin-orbit coupling operator) and for the g-tensor the magnetic field was included in the free-particle Foldy-Wouthuysen transformation in the DKH protocol (fπFW). Relativitically recontracted **DKH-DEF2-TZVP** basis set were chosen for all the atoms (auxiliary basis-sets were generated automatically − AutoAux). The g and spin-orbit contribution to the zero-field splitting tensor were computed using the effective Hamiltonian approach ^[27-29]^. The splitting of the d orbitals was computed using ab-initio ligand field analysis in the ORCA code ^[30]^. The calculation of the Fermi-contact and Spin-Dipolar contributions to the hyperfine tensors was carried out using **DLPN0-CCSD**^[31-37]^, following Jaworski and Hedin ^[38]^, considering unrelaxed coupled cluster density, Λ-equations, and quasi-restricted orbitals (t1 diagnostic was 0.0140 and 0.0143 for the low-spin and high-spin configurations, respectively). The **aug-cc-pVTZ-J**^[39]^ basis set along with aug-cc-pwCVTZ/C ^[40,41]^ auxiliary correlation fitting basis set was employed for the Mn atom while for the remaining ligand atoms, the smaller **EPR-II** basis-sets ^[42-45]^, combined with the auxiliary **cc-pwCVDZ/C** basis-sets, were chosen. **def2/JK** auxiliary basis-set was chosen for coulomb fitting. The calculation of the second-order spin-orbit perturbative corrections to the hyperfine tensor were calculated at DFT level of theory using the **B3PW91** functional ^[8]^, following Gomez-Piñeiro et al ^[46]^. The spin-orbit coupling operator was treated by the SOMF approximation, including the one-electron terms, the Coulomb terms in a semi-numeric way, incorporate exchange via one center exact integrals, the spin-other orbit interaction and include local DFT correlation. The basis-sets were kept the same as the calculation of the Fermi-Contact and Spin-Dipolar contributions to the A tensor. Orbital chemical shielding tensors were calculated at the meta-GGA level of theory using the **TPSS** functional ^[47]^ with **pcSseg-2**^[48]^ (def2/JK auxiliary basis-set) for all atoms and employing the **Split-RI-J** Coulomb approximation and the GIAO formalism ^[49-51]^. The NMR shielding tensors, $\sigma_{calc}$, were converted to shift convention according to:

$\delta= \sigma_{calc,ref} - \sigma_{exp,ref}$ (S1)

where $\sigma_{exp,ref}$ and $\sigma_{calc,ref}$correspond to the experimental and calculated isotropic shielding of a chosen reference compound, and $\sigma_{calc}$ = $\sigma_{dia}$ + $\sigma_{para}$, as explained in the main article. The reference shieldings were calculated at the same level of theory as the [MnL1]^+^ molecular clusters. Gas phase CH_4_ (^1^H and ^13^C) ^[52]^ were chosen as reference compounds. The calculation of the contribution of the paramagnetism of the unpaired electrons was calculated using the **pnmr** module in the ORCA code ^[6]^.

**Modern Kurland McGarvey formalism**

As mentioned in the main manuscript, within the recent formulation of the Kurland-McGarvey theory ^[53-55]^ the shielding tensor can be written as:

$\boldsymbol{\sigma}= \boldsymbol{\sigma}_{orb} -\frac{\mu_{B}}{\hbar\gamma_{K}kT} \boldsymbol{g}\cdot\left\langle\boldsymbol{SS} \right\rangle\cdot\boldsymbol{A}$ (S2)

Within this expression four terms are calculated, with $\sigma_{orb}$ being the relatively temperature independent diamagnetic orbital shielding term and is obtained via DFT calculation of the optimized structure, with the use of the TPSS functional. The ***g-*** and ***A-***tensors as well as the Spin dyadic $\left\langle\boldsymbol{SS} \right\rangle$ can be further broken down as well. In particular, the ***g-***tensor can be decomposed into:

$\boldsymbol{g}=\boldsymbol{g}_{e}+{\boldsymbol{\Delta}\boldsymbol{g}}_{iso}+ \boldsymbol{\Delta}\tilde{\boldsymbol{g}}$ (S3)

Here, $\boldsymbol{g}_{e}$ corresponds to the free-electron g-tensor, ${\boldsymbol{\Delta}\boldsymbol{g}}_{iso}$ is the isotropic deviation and $\boldsymbol{\Delta}\tilde{\boldsymbol{g}}$ the traceless anisotropic term. The ***A-***tensor is depicted by:

$\boldsymbol{A}=A_{con}\boldsymbol{1}+\mathbf{A}_{dip}+ A_{pc}\boldsymbol{1}+\boldsymbol{A}_{\boldsymbol{dip,2}}+ \boldsymbol{A}_{as}$ (S4)

with **1** being a 3x3 unit matrix, $A_{con}$and $\mathbf{A}_{\boldsymbol{dip}}$ the isotropic fermi-contact and anisotropic dipolar parts, respectively that are non-relativistic; the relativistic terms comprise the isotropic pseudocontact $A_{pc}$, the symmetric 2^nd^ order dipolar term $\boldsymbol{A}_{\boldsymbol{dip,2}}$ and the antisymmetric term $\boldsymbol{A}_{as}$.

The Spin dyadic that the hyperfine and g-tensor operate with can be written as follows:

$\left\langle S_{u}S_{v} \right\rangle= \frac{\sum_{mn} Q_{mn}\left\langle n | S_{u} | m \right\rangle\left\langle m | S_{v} | n \right\rangle}{\sum_{n} exp\left( \frac{-E_{n}}{kT} \right)}$ (S5)

with $Q_{mn}$ being:

$Q_{mn}= \left\{ \begin{aligned} \exp\left( -\frac{E_{n}}{kT} \right) E_{n}=E_{m} \\ -\frac{kT}{E_{m}-E_{n}}\left[ \exp\left( -\frac{E_{m}}{kT} \right)-\exp\left( -\frac{E_{n}}{kT} \right) \right] E_{n} \neq E_{m} \end{aligned} \right.$ (S6)

i.e., the thermal average over the zero-field split ground state multiplet of $\left. n \right\rangle$ eigenfunctions and $E_{n}$ eigenvalues of the Zero Field splitting Hamiltonian.

From the expression of the A- and g-tensor components, together with the ZFS operator, the overall pNMR shift can be expressed as a 9 distinct shift contributions^[38,55]^_:_

$\boldsymbol{\sigma}=\sigma_{orb}+\sigma_{con}+ \sigma_{con,2}+\sigma_{con,3}+ \sigma_{dip}+ \sigma_{dip,2}+ \sigma_{dip,3}+ \sigma_{c,aniso}+ \sigma_{pc}$ (S7)

The 1^st^ term, $\sigma_{orb},$ corresponds to the diamagnetic orbital shielding, $\sigma_{con}$, $\sigma_{con,2}$ and $\sigma_{con,3}$ correspond to the Fermi-Contact contribution, whereas the remaining terms correspond to the pseudo contact shift. Additionally, by taking into account the PSO correction we obtain the complete pNMR shielding tensor.

The isotropic average of the pNMR shielding tensor is:

$\sigma_{iso}= \frac{\sigma_{xx} + \sigma_{yy}+ \sigma_{zz}}{3}$ (S8)

**Results and Discussion**

**
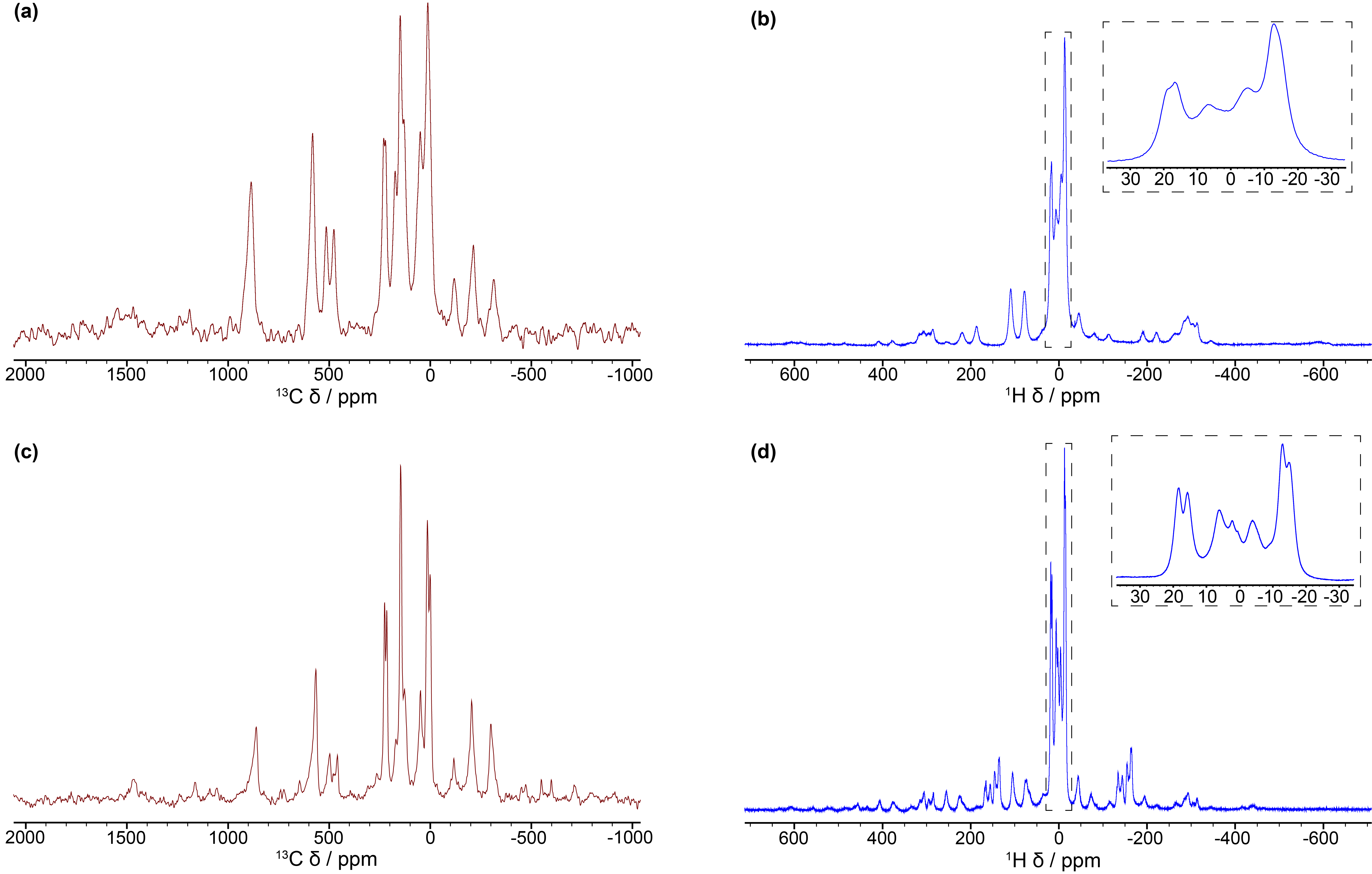
**

**Figure S2.** Comparison of the 1-D ssNMR spectra recorded at 4.7T and 9.4T at a spinning speed of 60 kHz. (a) ^13^C and (b) ^1^H are the 1-D solid-state NMR spectra of MnL1^+^[PF_6_]^-^ at 4.7T that were recorded with a rotor-synchronized Hahn-echo sequence. (c) and (d) are the respective ssNMR spectra acquired with a rotor-synchronized double adiabatic echo sequence at 9.4T. The inserts in (b) and (d) highlight the spectral region between -15 and 30 ppm. In contrast to signals outside of this spectral region, linewidths of these signals are not dominated by inhomogeneous broadening, as they are less influenced by the paramagnetic effects mentioned in the main manuscript. Moreover, by comparing the spectra at the two magnetic fields for both nuclei, it becomes evident that the scaling of the anisotropy (in Hz) with respect to the magnetic field, leads to significant spinning-sideband overlap.


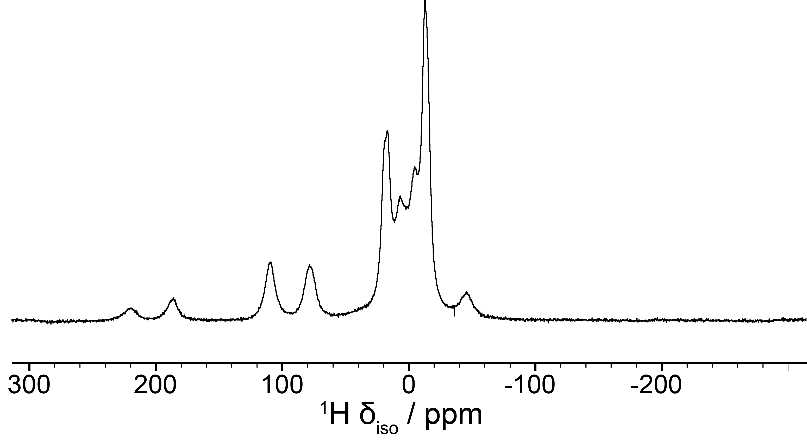


**Figure S3**. The isotropic ^1^H projection MATPASS NMR spectrum of MnL1^+^[PF_6_]^-^ recorded at a magnetic field strength of 4.7 T and spinning speed of 60 kHz. Compared to the 1-D spectrum in Figure S2b, the use pj-MATPASS sequence enables the identification of both the signal at 221 ppm and the negative shift at -45 ppm which would otherwise overlap.


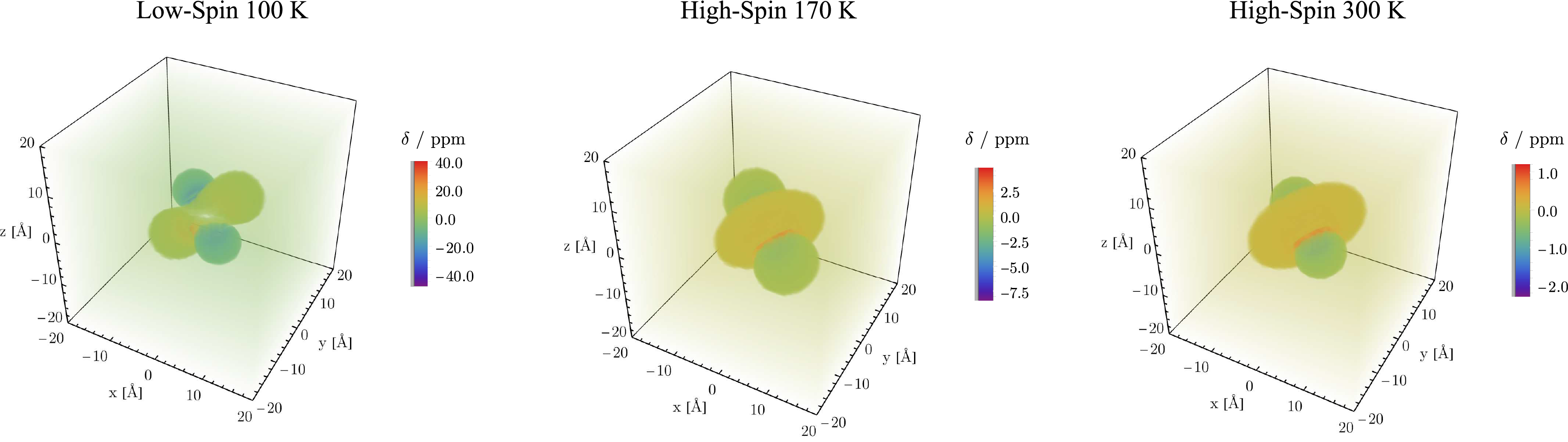


**Figure S4**. The magnitude of inter-molecular pseudocontact shift by use of the point-dipole approximation is negligible for both the LS state (100K) and HS state at 170K and 300K, which are the relevant experimental temperatures.


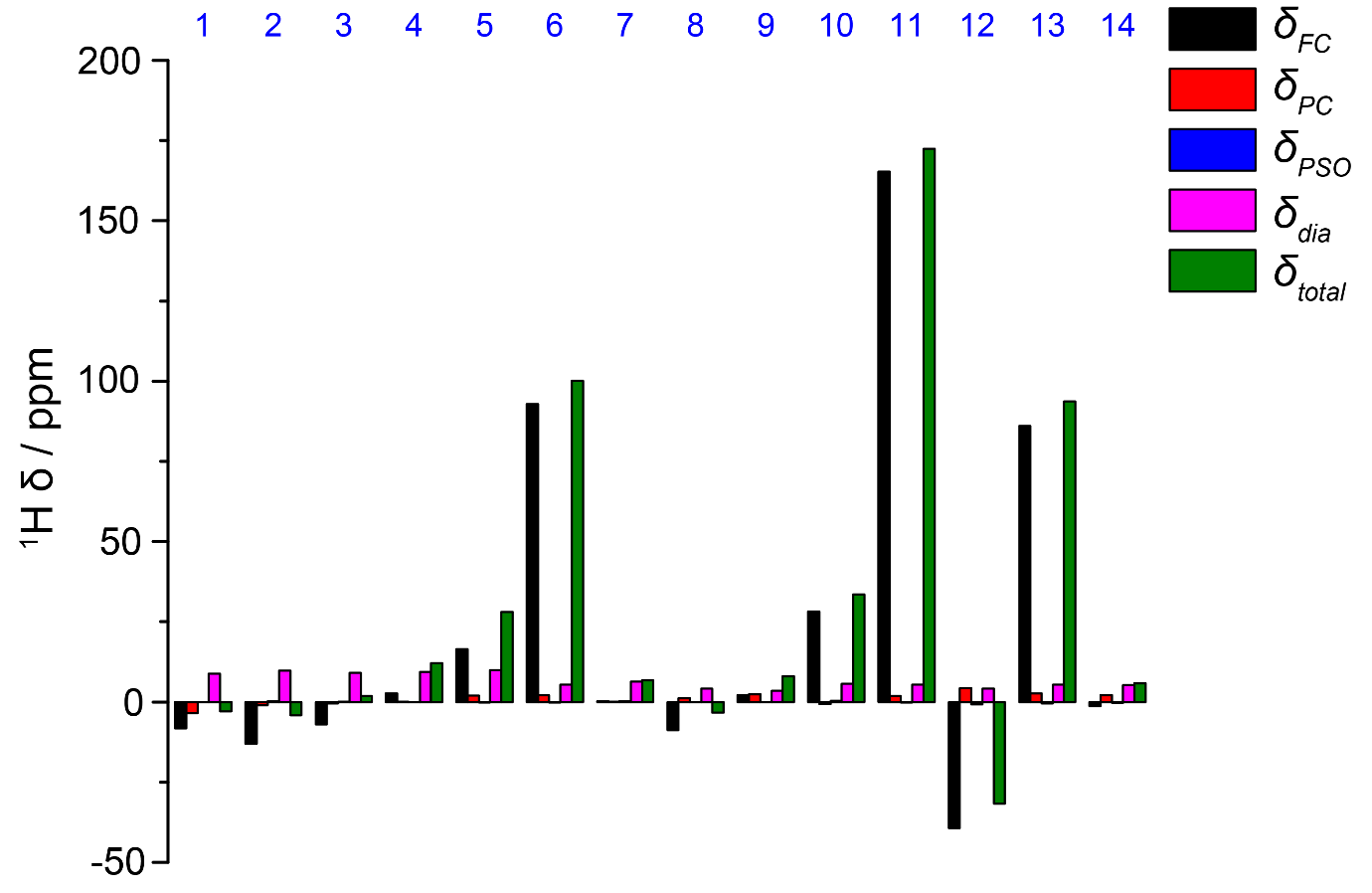


**Figure S5.** The ^1^H shift contribution histograms for each crystallographically inequivalent site at specific temperatures at 320K.

**
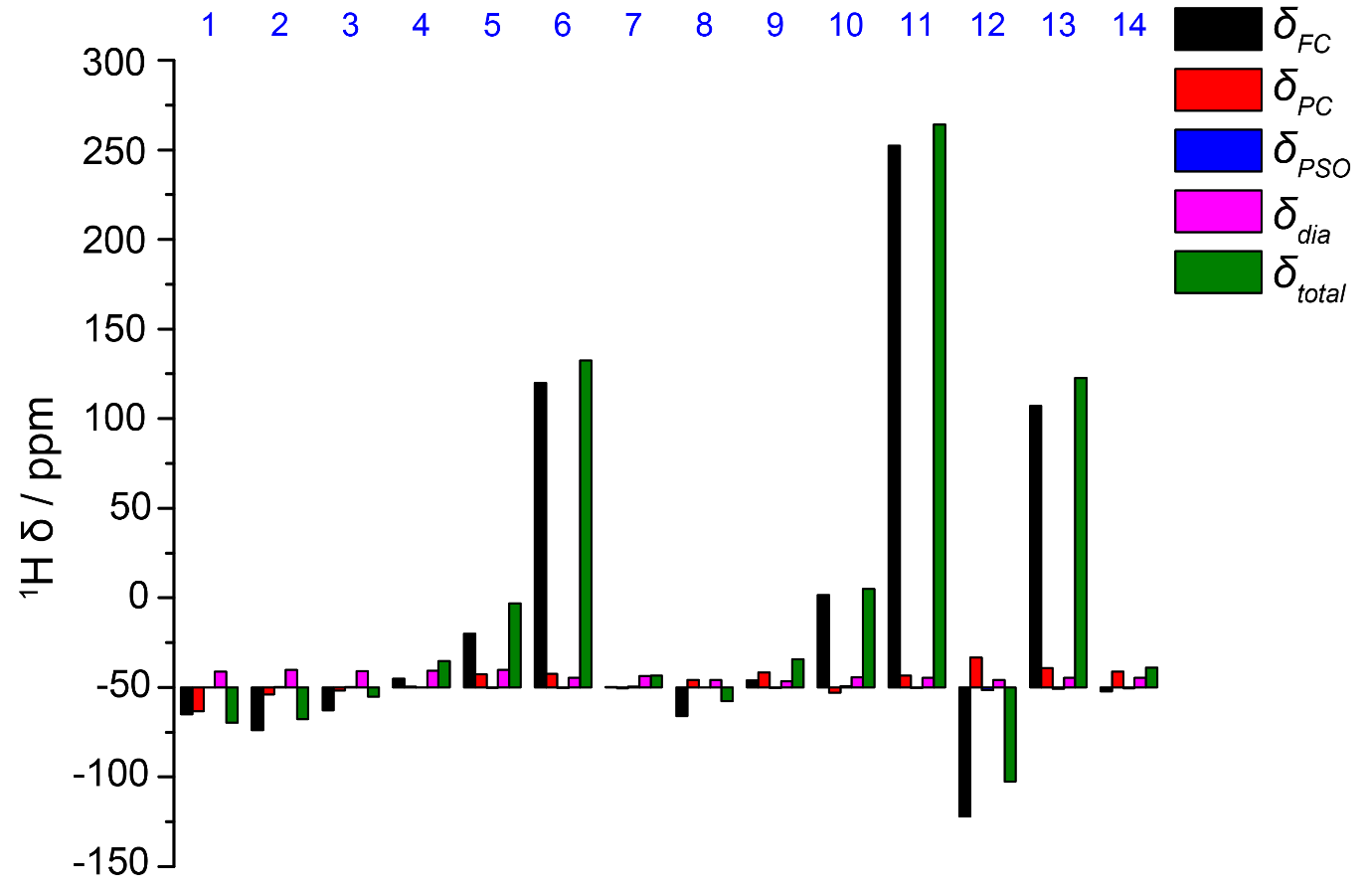
**

**Figure S6.** The ^1^H shift contribution histograms for each crystallographically inequivalent site at specific temperatures at 175K.


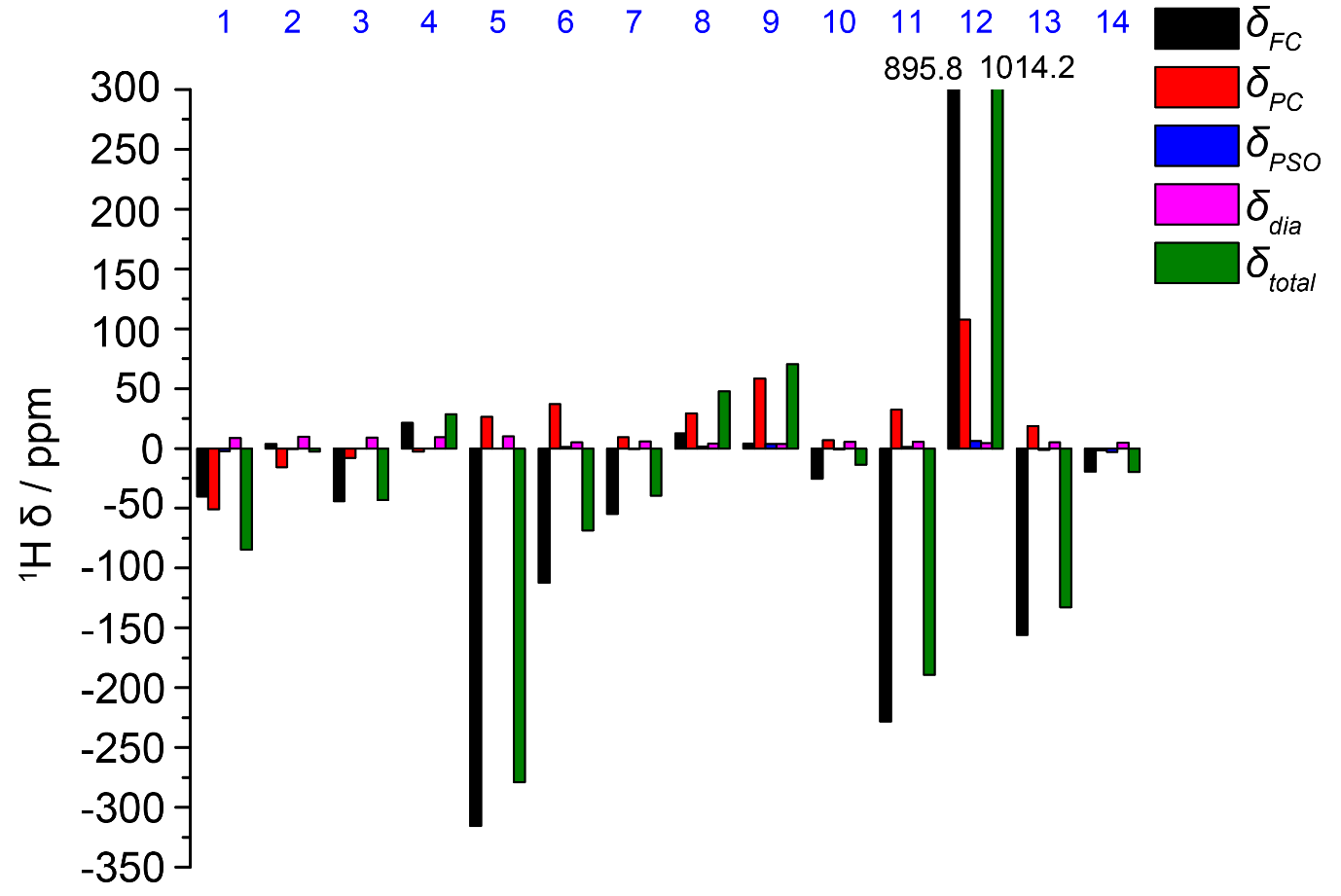


**Figure S7.** The ^1^H shift contribution histograms for each crystallographically inequivalent site at specific temperatures at 105K.


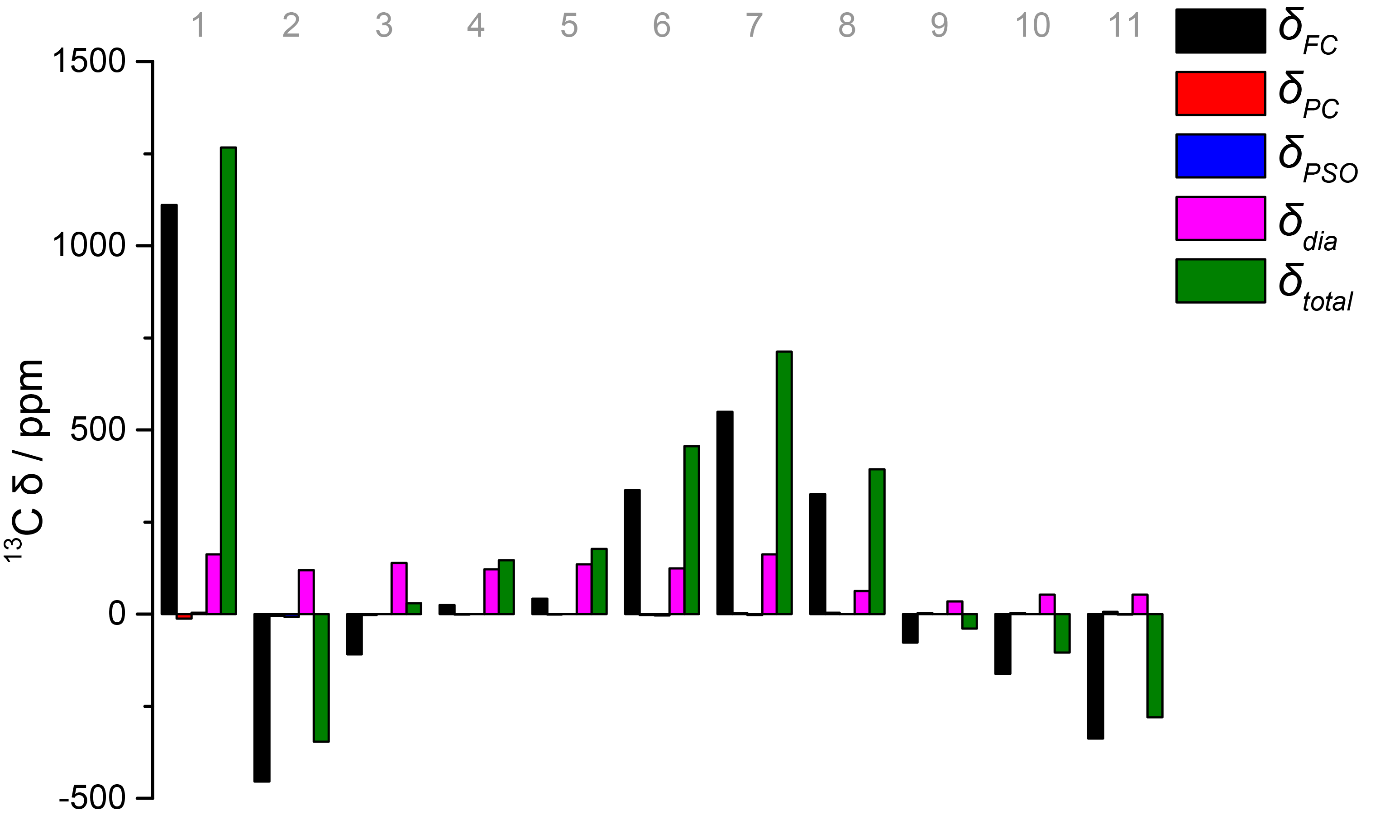


**Figure S8.** The ^13^C shift contribution histograms for each crystallographically inequivalent site at specific temperatures at 320K.


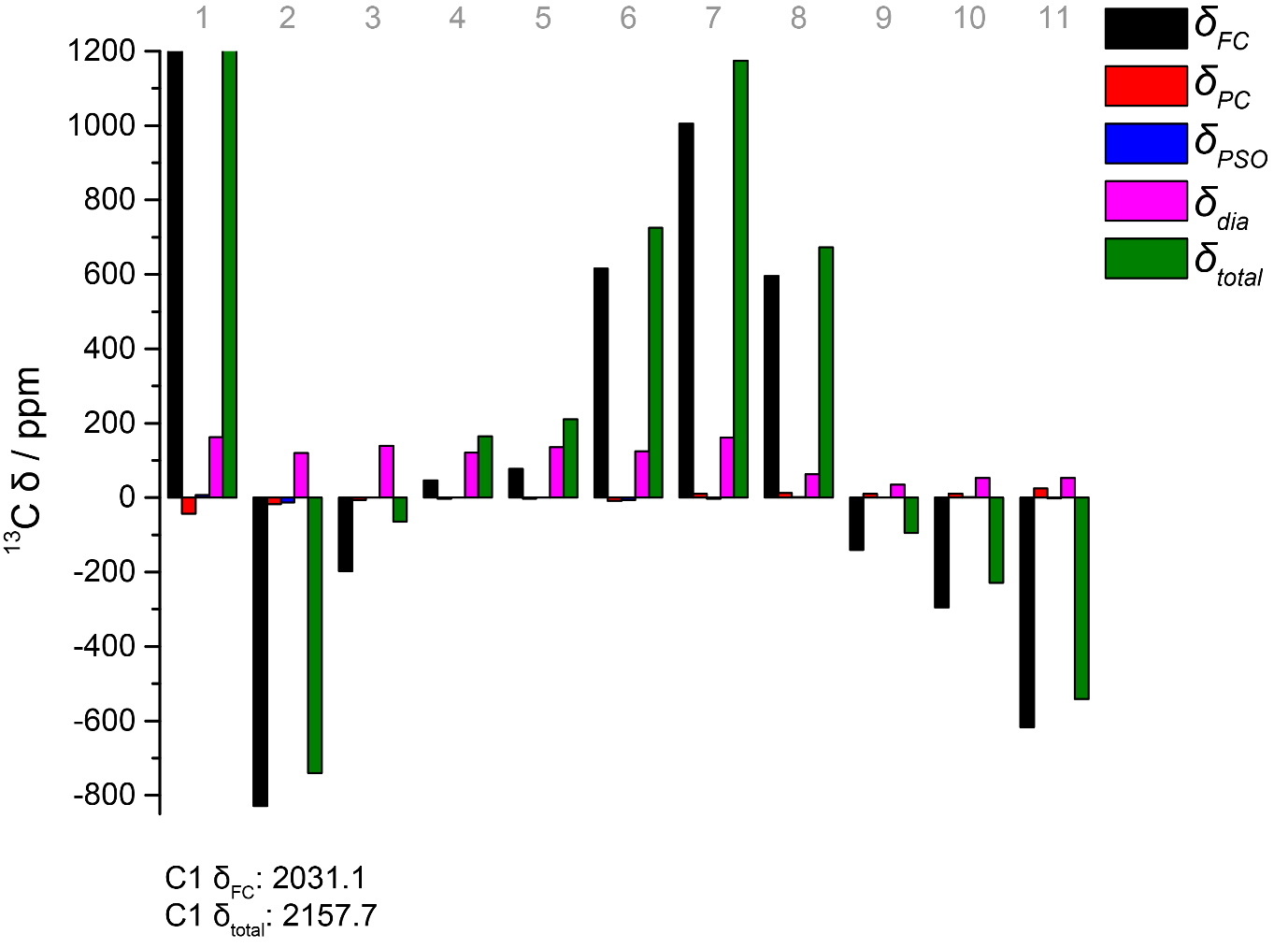


**Figure S9.** The ^13^C shift contribution histograms for each crystallographically inequivalent site at specific temperatures at 175K.


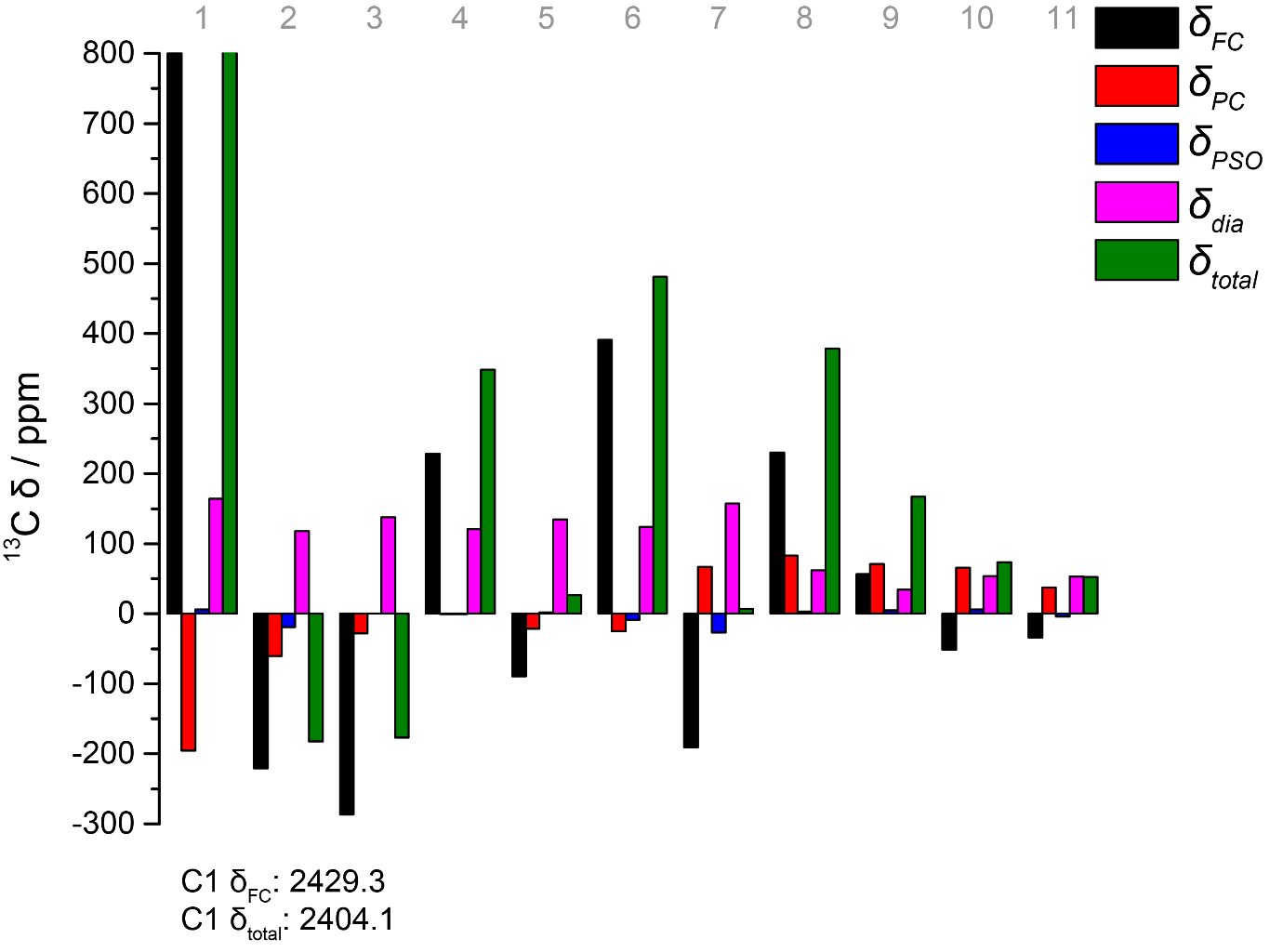


**Figure S10.** The ^13^C shift contribution histograms for each crystallographically inequivalent site at specific temperatures at 105K.


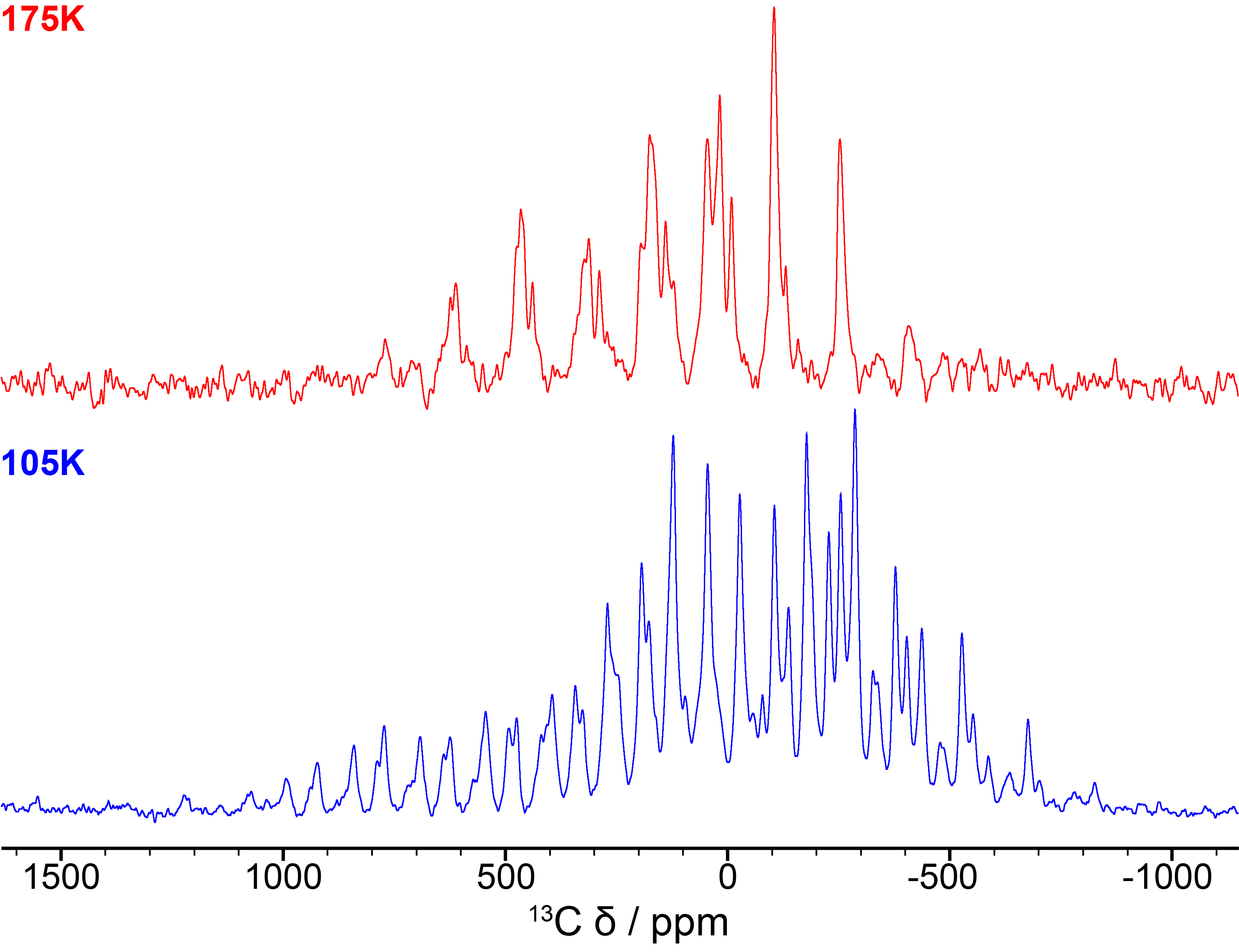


**Figure S11.** The 1-D ^13^C double adiabatic echo spectra that were acquired at 105K (LS) and 175K (HS). The intensity of the HS spectrum has been scaled up 6 times to match the number of scans accumulated for the acquisition of the LS spectrum.


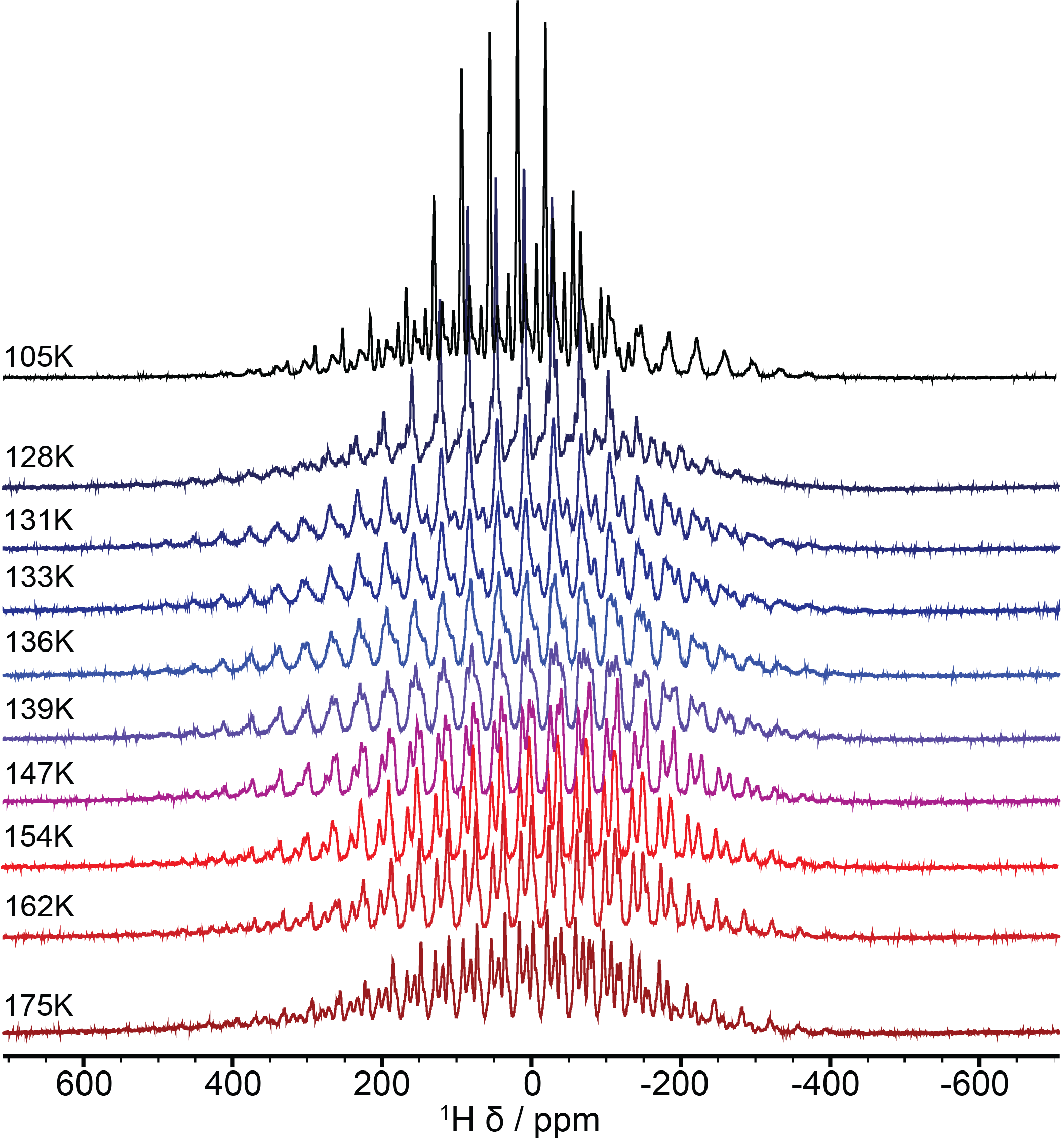


**Figure S12**. The 1-D LTMAS NMR spectra at temperatures that highlight the pure spin states (the LS state at 105 K, and the HS state at 175 K), as well as temperatures around the spin transition temperature $T_{ST}$ (128 K to 162 K). The shift anisotropy increases around the spin transition and at the same time, the signal intensity drops by a factor of ~2.


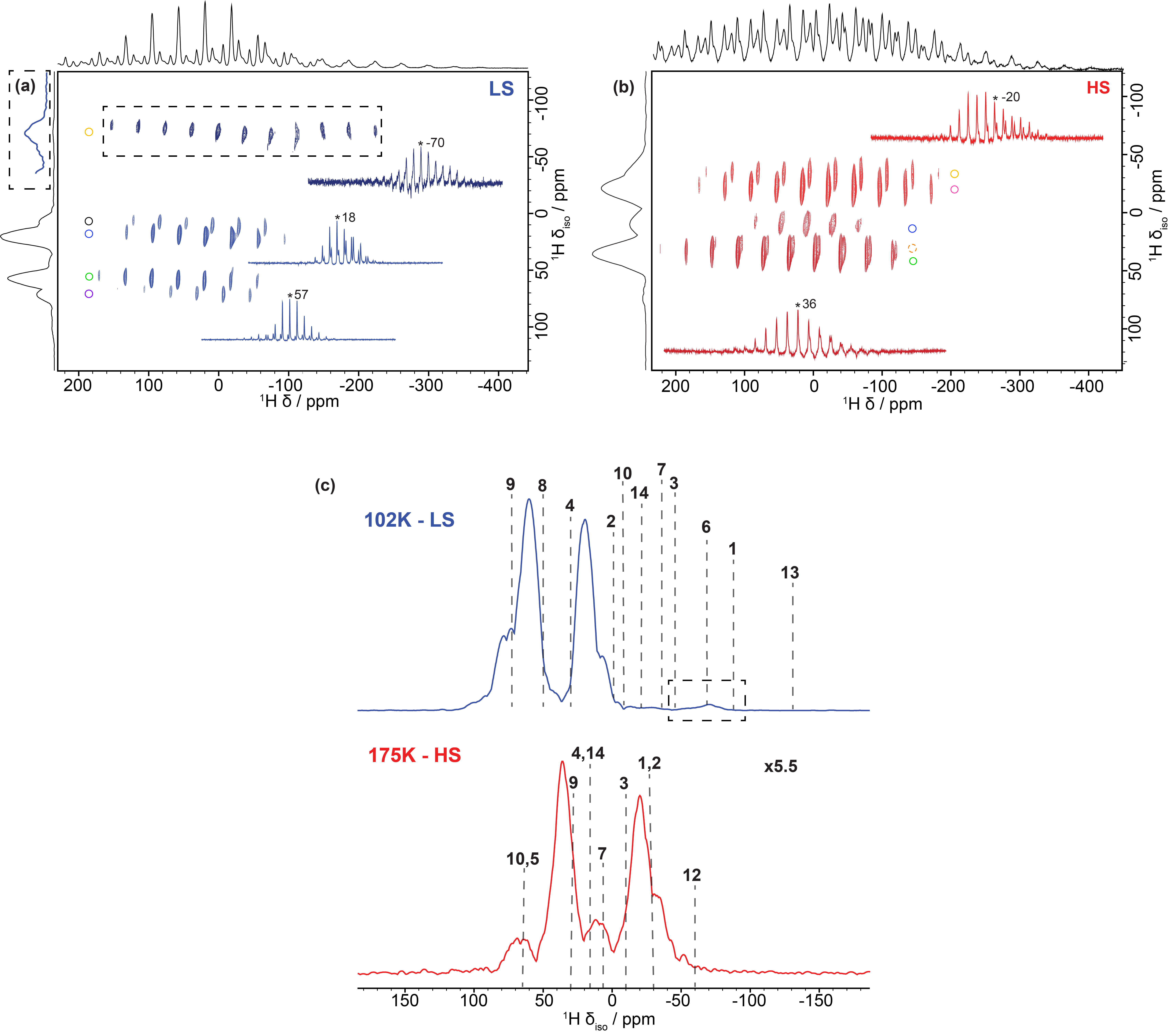


**Figure S13. Detection of the LS and HS states with ^1^H aMAT NMR.** **a,b** 2D ^1^H aMAT NMR spectra at 30 kHz MAS in magnetic field of 18.8 T. The left panel (3a in blue color) is at 102 K (LS state), and the right panel (3b in red color) at 175 K (HS state). The 1D insets in both spectra are cross sections at the indicated δ_iso_, illustrating the relevant spinning sideband manifolds. On the top and left sides of the spectra are the relevant spectra projections. The colored circles are used as a guide **c.** The experimental ^1^H aMAT NMR projections in the isotropic δ_iso_ (vertical) direction at temperatures 102 K (LS), and 175 K (HS). The dashed lines correspond to the calculated isotropic shifts of the inequivalent ^1^H sites.


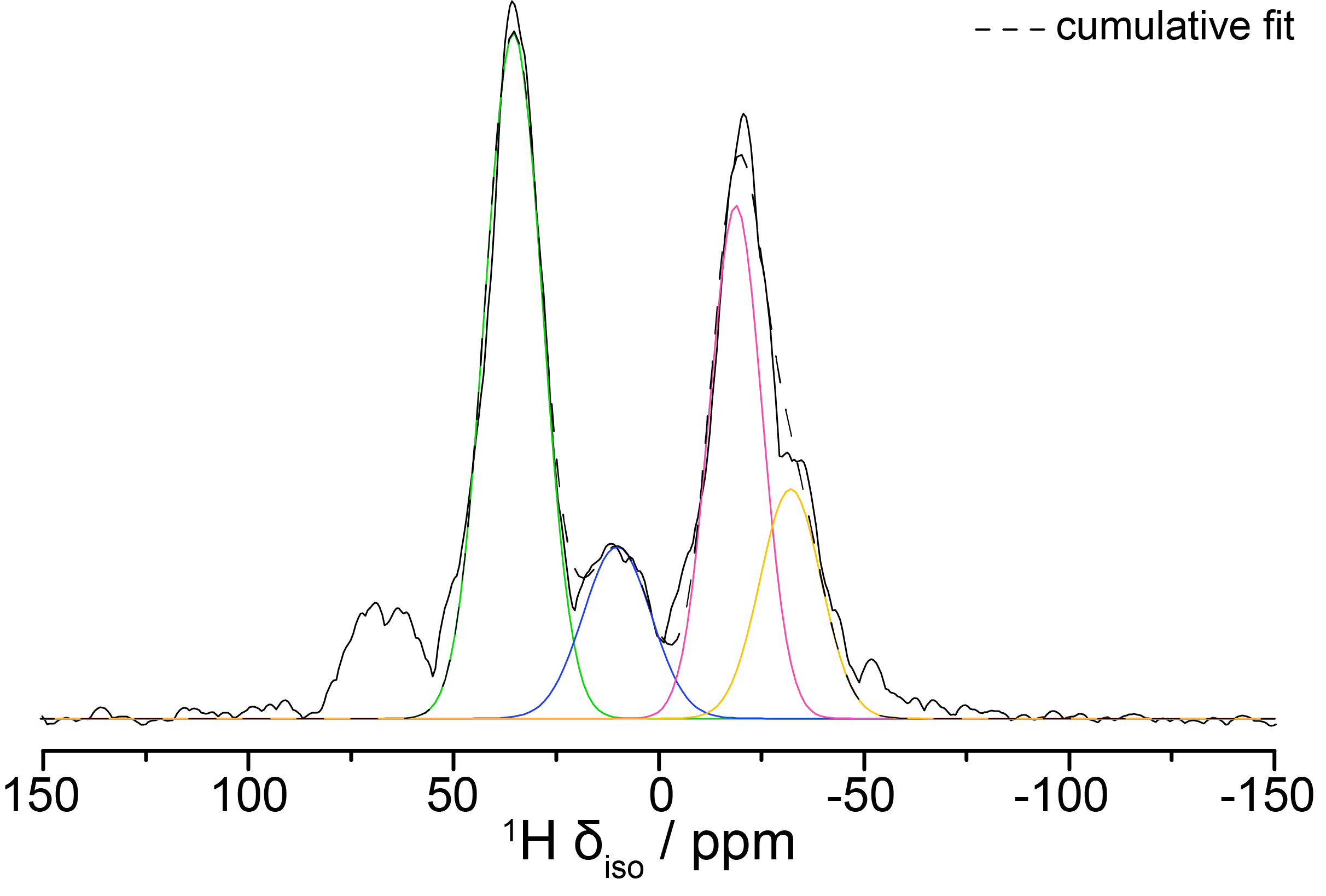


**FigureS14**. Deconvolution of the ^1^H indirect (isotropic) dimension via multi-gaussian fitting at 175K. No isotropic shift was detected in the diagonal of the respective 2-D spectrum at 71 ppm and thus this peak is considered an artifact.

**
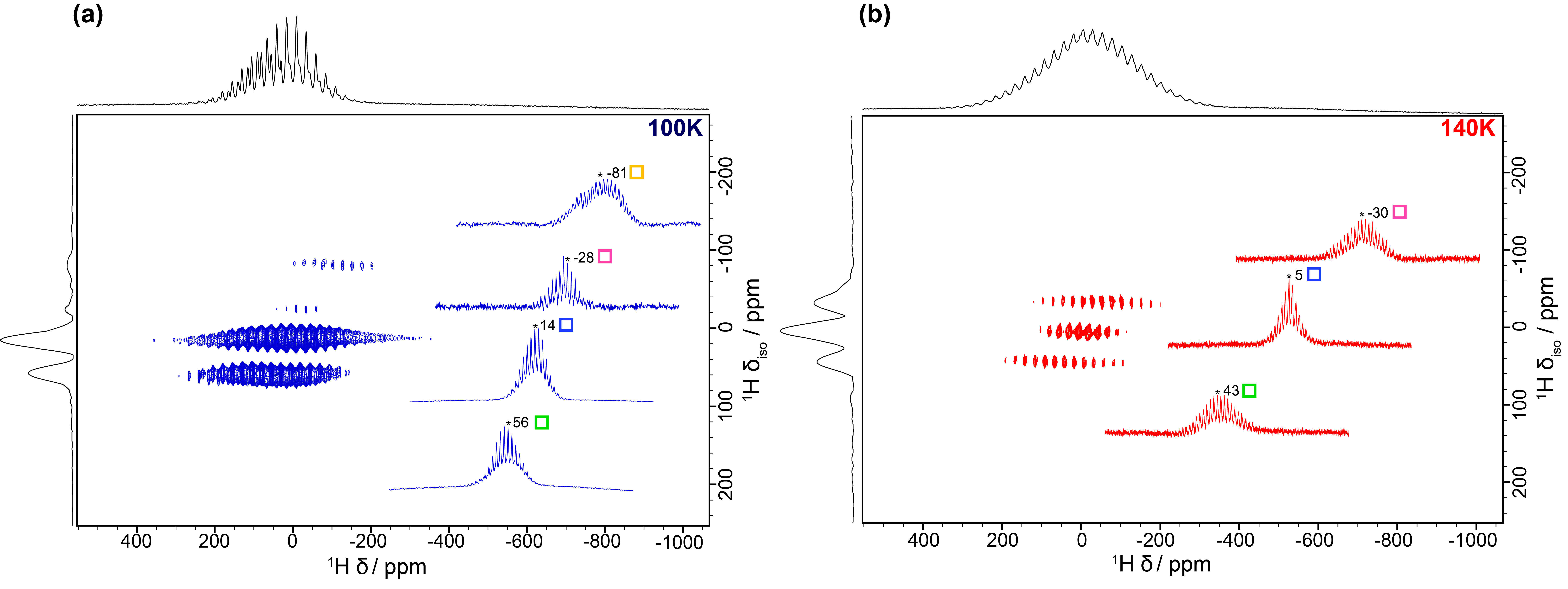
**

**Figure S15**. 2-D Magic Angle Turning (MAT) ^1^H NMR spectra at **(a)** 100 K (Low Spin state) and **(b)** 140 K (High Spin state) recorded at a magnetic field of 9.4 T and spinning speed of 10 kHz. The weak peak at -25 ppm in the LS state is invisible with the aMAT pulse sequence recorded at 18.8 T and 30 kHz, as described in Figure 5c of the main article, due to its extremely short transverse relaxation rate. The colored hollow rectangles are used as a color code for the isotropic shift evolution and intensity in Figures 5c of the main manuscript (in the LS regime) and Figures S16, S17.


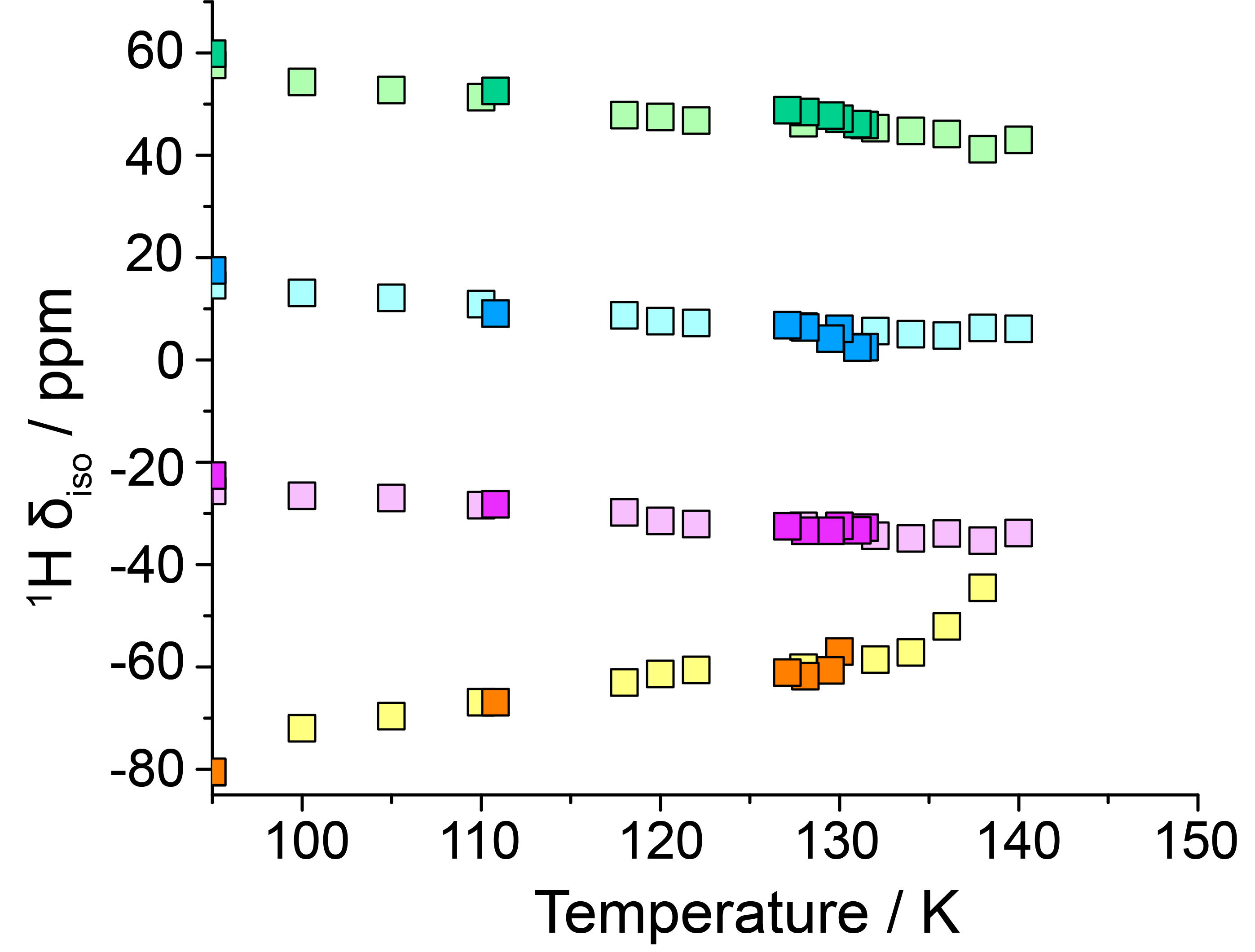


**Figure S16**. Frequency shift evolution of the isotropic ^1^H MAT NMR projections in the temperature range 95-140K at 9.4 T and MAS frequency of 10 kHz. Light colored rectangles denote heating mode whereas dark colored rectangles denote the cooling mode.


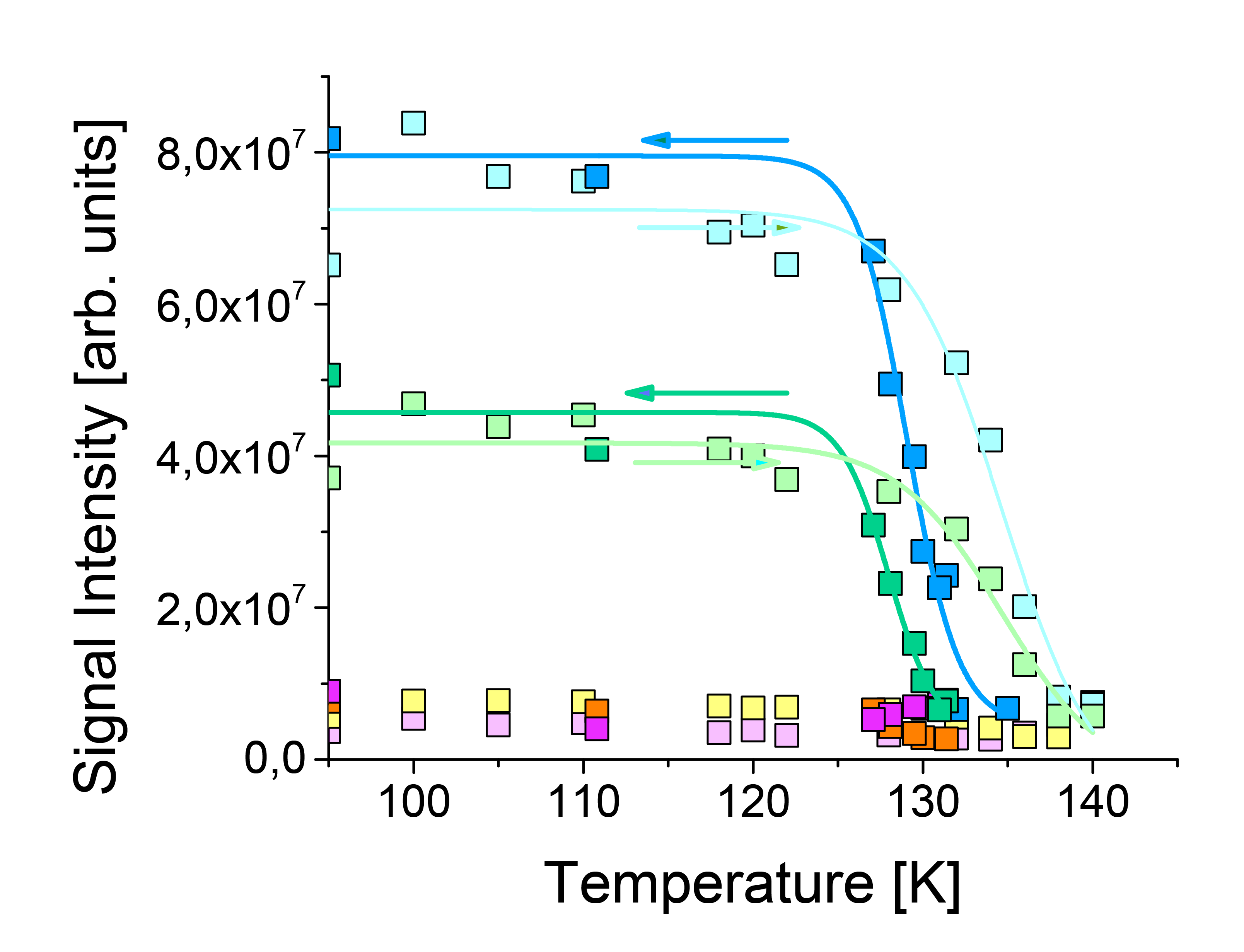


**Figure S17**. Signal intensity vs. temperature of the isotropic ^1^H MAT NMR, light colored rectangles denote heating mode whereas dark colored rectangles denote the cooling mode. A hysteresis loop can be identified in the case of the shifts corresponding to the blue and green rectangles (**H9** and **H4** according to calculations, respectively).

**Table S1.** The calculated hyperfine coupling constants (HFCC) for the 14 inequivalent atom sites in both spin states. This is term is scalar to the transferred electron spin density on the nucleus. The values of both spin-states are of similar magnitude, except **H5** and **H12** in the LS state.

| **H atoms** | **A_iso_ – HS (MHz)** | **A_iso_ – LS (MHz)** |
| --- | --- | --- |
| 1 | -0.0420 | -0.1973 |
| 2 | -0.0657 | 0.0154 |
| 3 | -0.0351 | -0.2075 |
| 4 | 0.0136 | 0.1008 |
| 5 | 0.0827 | -1.4727 |
| 6 | 0.4729 | -0.5194 |
| 7 | 0.0019 | -0.2583 |
| 8 | -0.0445 | 0.0646 |
| 9 | 0.0104 | 0.0343 |
| 10 | 0.1452 | -0.1236 |
| 11 | 0.8429 | -1.0615 |
| 12 | -0.2049 | 4.2076 |
| 13 | 0.4365 | -0.7316 |
| 14 | -0.0082 | -0.1032 |

**Table S2.** The calculated ^13^C HFCCs for both spin-states.

| **C atoms** | **A_iso_ – HS (MHz)** | **A_iso_ – LS (MHz)** |
| --- | --- | --- |
| 1 | 1.4301 | 2.8562 |
| 2 | -0.5912 | -0.2796 |
| 3 | -0.1384 | -0.3364 |
| 4 | 0.0330 | 0.2768 |
| 5 | 0.0547 | -0.1026 |
| 6 | 0.4277 | 0.4490 |
| 7 | 0.7028 | -0.2495 |
| 8 | 0.419 | 0.2732 |
| 9 | -0.0979 | 0.0712 |
| 10 | -0.2062 | -0.0544 |
| 11 | -0.4369 | -0.0438 |

**Table S3.** The DFT optimized High-Spin structure (x,y,z in Å), used for the quantum chemical calculations of the EPR parameters and the paramagnetic NMR shifts.

Mn1 2.78915 0.25701 16.89629

O1 1.57414 1.31822 17.84123

C1 0.63501 0.92268 18.70324

C2 0.20819 1.82828 19.70435

H1 0.66872 2.81959 19.73587

C3 -0.78074 1.46436 20.61992

H2 -1.08613 2.18078 21.38842

C4 -1.39139 0.19461 20.56460

H3 -2.16473 -0.07906 21.28579

C5 -1.00723 -0.69785 19.56935

H4 -1.48701 -1.67959 19.49419

C6 0.00494 -0.36390 18.63267

C7 0.28345 -1.27386 17.55244

H5 -0.42525 -2.11322 17.43012

N1 1.25881 -1.16289 16.69059

C8 1.24785 -2.03361 15.51000

H6 0.52235 -2.85615 15.65603

H7 2.24923 -2.47667 15.38719

C9 0.86854 -1.23914 14.24593

H8 0.74787 -1.95935 13.41816

H9 -0.12318 -0.77192 14.39535

C10 1.88942 -0.18862 13.79872

H10 2.88164 -0.65711 13.68403

H11 1.59038 0.21021 12.80827

N2 2.05514 0.91955 14.76991

H12 1.13485 1.33692 14.96304

C11 2.95988 1.97507 14.26895

H13 2.54259 2.49949 13.38712

H14 3.89201 1.47899 13.94386

C12 3.26198 2.97294 15.38303

H15 3.87907 3.80626 14.99378

H16 2.32182 3.41167 15.76266

N3 3.91000 2.28092 16.51622

H17 4.84545 1.96844 16.22328

C13 4.04110 3.15253 17.70889

H18 3.01547 3.38937 18.03978

H19 4.53388 4.10719 17.43467

C14 4.81816 2.49785 18.85483

H20 4.94242 3.25566 19.64778

H21 5.83866 2.22957 18.52198

C15 4.15074 1.26065 19.48436

H22 4.71655 0.95939 20.38631

H23 3.12114 1.50577 19.79098

N4 4.10649 0.14912 18.52789

C16 4.92947 -0.85041 18.70144

H24 5.51763 -0.88329 19.63664

C17 5.18829 -1.90364 17.75459

C18 6.00069 -2.99782 18.14984

H25 6.33809 -3.04983 19.19059

C19 6.36677 -3.98820 17.24457

H26 6.98334 -4.83105 17.56482

C20 5.94485 -3.88089 15.90347

H27 6.23982 -4.64554 15.17864

C21 5.15806 -2.80841 15.47976

H28 4.84642 -2.71547 14.43572

C22 4.74831 -1.80691 16.39268

O2 4.00483 -0.79468 15.94015

**Table S4.** The DFT optimized Low-Spin structure (x,y,z in Å), used for the quantum chemical calculations of the EPR parameters and the paramagnetic NMR shifts.

Mn1 2.80825 0.39645 16.75979

O1 1.48325 1.47230 17.55046

C1 0.64987 1.09988 18.52214

C2 0.09045 2.08761 19.37045

H1 0.36552 3.13259 19.20155

C3 -0.78810 1.73049 20.39379

H2 -1.19885 2.50998 21.04242

C4 -1.15826 0.38344 20.59777

H3 -1.85026 0.11699 21.39996

C5 -0.64904 -0.59594 19.75276

H4 -0.94907 -1.64206 19.87625

C6 0.26475 -0.26858 18.71636

C7 0.64606 -1.26905 17.75941

H5 0.03577 -2.18678 17.73466

N1 1.59157 -1.14372 16.85602

C8 1.55824 -2.08383 15.72389

H6 0.92754 -2.95306 15.98420

H7 2.57331 -2.45461 15.51640

C9 0.98039 -1.38660 14.47874

H8 0.85842 -2.14139 13.68274

H9 -0.03279 -1.00754 14.70875

C10 1.85920 -0.26629 13.91846

H10 2.86882 -0.65557 13.70987

H11 1.43309 0.11075 12.96908

N2 2.04105 0.86699 14.86650

H12 1.12385 1.27710 15.09328

C11 2.92154 1.92965 14.30731

H13 2.44541 2.44933 13.45558

H14 3.82976 1.42734 13.93320

C12 3.27883 2.91499 15.41303

H15 3.94711 3.70910 15.03230

H16 2.36763 3.39835 15.80482

N3 3.89492 2.17444 16.54906

H17 4.82311 1.83205 16.26222

C13 4.03197 3.02693 17.76167

H18 3.01411 3.36488 18.01531

H19 4.63968 3.91962 17.51918

C14 4.64255 2.28647 18.95336

H20 4.74841 3.01399 19.77655

H21 5.66352 1.93674 18.71171

C15 3.80337 1.09904 19.46076

H22 4.25362 0.70090 20.38813

H23 2.78080 1.42971 19.69715

N4 3.76041 0.03881 18.44095

C16 4.54732 -0.99703 18.62534

H24 5.01435 -1.11239 19.61714

C17 4.93516 -1.94314 17.61665

C18 5.63767 -3.12056 17.98640

H25 5.76502 -3.34258 19.05133

C19 6.15571 -3.98042 17.02471

H26 6.68184 -4.89049 17.32191

C20 6.01319 -3.65348 15.65877

H27 6.43400 -4.31517 14.89578

C21 5.34919 -2.49192 15.26329

H28 5.25212 -2.22918 14.20604

C22 4.78071 -1.62210 16.22671

O2 4.15438 -0.51824 15.81753

**References**

1. P. N. Martinho, B. Gildea, M. M. Harris, T. Lemma, A. D. Naik, H. Müller-Bunz, T. E. Keyes, Y. Garcia, G. G. Morgan, Angew. Chem. Int. Ed. **2012**, 51, 12597–12601.
2. G. Kervern, G. Pintacuda, L. Emsley, Chemical Physics Letters **2007**, 435, 157–162.
3. M. Garwood, L. DelaBarre, Journal of Magnetic Resonance **2001**, 153, 155–177.
4. Z. Gan, J. Am. Chem. Soc. **1992**, 114, 8307–8309.
5. K. R. Thurber, R. Tycko, J. Magn. Reson. 2009, 196, 84–87.
6. F. Neese, F. Wennmohs, U. Becker, C. Riplinger, The Journal of Chemical Physics **2020**, 152, 224108.
7. J. P. Perdew, M. Ernzerhof, K. Burke, The Journal of Chemical Physics **1996**, 105, 9982–9985.
8. A. D. Becke, The Journal of Chemical Physics **1993**, 98, 5648–5652.
9. C. Adamo, V. Barone, The Journal of Chemical Physics **1999**, 110, 6158–6170.
10. E. Caldeweyher, S. Ehlert, A. Hansen, H. Neugebauer, S. Spicher, C. Bannwarth, S. Grimme, J. Chem. Phys. **2019**, 150, 154122.
11. F. Weigend, R. Ahlrichs, Phys. Chem. Chem. Phys. **2005**, 7, 3297.
12. F. Weigend, J. Comput. Chem. **2008**, 29, 167–175.
13. P. Siegbahn, A. Heiberg, B. Roos, B. Levy, Phys. Scr. **1980**, 21, 323–327.
14. B. O. Roos, P. R. Taylor, P. E. M. Siegbahn, Chem. Phys. **1980**, 48, 157–173.
15. P. E. M. Siegbahn, J. Almlöf, A. Heiberg, B. O. Roos, J. Chem. Phys. **1981**, 74, 2384–2396.
16. C. Angeli, R. Cimiraglia, S. Evangelisti, T. Leininger, J.-P. Malrieu, J. Chem. Phys. **2001**, 114, 10252–10264.
17. C. Angeli, R. Cimiraglia, J.-P. Malrieu, J. Chem. Phys. **2002**, 117, 9138–9153.
18. C. Angeli, M. Pastore, R. Cimiraglia, Theor. Chem. Acc. **2007**, 117, 743–754.
19. M. Douglas, N. M. Kroll, Ann. Phys. **1974**, 82, 89–155.
20. B. A. Hess, Phys. Rev. A **1986**, 33, 3742–3748.
21. M. Reiher, Theor. Chem. Acc. **2006**, 116, 241–252.
22. D. Ganyushin, F. Neese, J. Chem. Phys. **2006**, 125, 024103.
23. D. Ganyushin, F. Neese, J. Chem. Phys. **2013**, 138, 104113.
24. B. A. Hess, C. M. Marian, U. Wahlgren, O. Gropen, Chem. Phys. Lett. **1996**, 251, 365–371.
25. F. Neese, J. Chem. Phys. **2005**, 122, 034107.
26. A. Pyykkönen, R. Feher, F. H. Köhler, J. Vaara, Inorg. Chem. **2020**, 59, 9294–9307.
27. F. Neese, Int. J. Quantum Chem. **2001**, 83, 104–114.
28. J.-P. Malrieu, R. Caballol, C. J. Calzado, C. De Graaf, N. Guihéry, Chem. Rev. **2014**, 114, 429–492.
29. M. Atanasov, et al., Coord. Chem. Rev. **2015**, 289–290, 177–214.
30. L. Lang, M. Atanasov, F. Neese, J. Phys. Chem. A **2020**, 124, 1025–1037.
31. C. Riplinger, F. Neese, J. Chem. Phys. **2013**, 138, 034106.
32. C. Riplinger, B. Sandhoefer, A. Hansen, F. Neese, J. Chem. Phys. **2013**, 139, 134101.
33. Y. Guo, C. Riplinger, U. Becker, D. G. Liakos, Y. Minenkov, L. Cavallo, F. Neese, The Journal of Chemical Physics **2018**, 148, 011101.
34. C. Riplinger, P. Pinski, U. Becker, E. F. Valeev, F. Neese, J. Chem. Phys. **2016**, 144, 024109.
35. M. Saitow, U. Becker, C. Riplinger, E. F. Valeev, F. Neese, J. Chem. Phys. **2017**, 146, 164105.
36. Y. Guo, C. Riplinger, D. G. Liakos, U. Becker, M. Saitow, F. Neese, The Journal of Chemical Physics **2020**, 152, 024116.
37. M. Saitow, F. Neese, J. Chem. Phys. **2018**, 149, 034104.
38. A. Jaworski, N. Hedin, Phys. Chem. Chem. Phys. **2022**, 24, 15230–15244.
39. E. D. Hedegård, J. Kongsted, S. P. A. Sauer, J. Chem. Theory Comput. **2011**, 7, 4077–4087.
40. F. Weigend, A. Köhn, C. Hättig, J. Chem. Phys. **2002**, 116, 3175–3183.
41. C. Hättig, Phys. Chem. Chem. Phys. **2005**, 7, 59–66.
42. D. P. Chong, Recent Advances in Density Functional Methods: (Part I), World Scientific, **1995**.
43. N. Rega, M. Cossi, V. Barone, J. Chem. Phys. **1996**, 105, 11060–11067.
44. N. Rega, M. Cossi, V. Barone, J. Am. Chem. Soc. **1997**, 119, 12962–12967.
45. N. Rega, M. Cossi, V. Barone, J. Am. Chem. Soc. **1998**, 120, 5723–5732.
46. R. J. Gómez-Piñeiro, D. A. Pantazis, M. Orio, ChemPhysChem **2020**, 21, 2667–2679.
47. J. Tao, J. P. Perdew, V. N. Staroverov, G. E. Scuseria, Phys. Rev. Lett. **2003**, 91, 146401.
48. F. Jensen, J. Chem. Theory Comput. **2015**, 11, 132–138.
49. F. London, J. Phys. Radium **1937**, 8, 397–409.
50. R. Ditchfield, Mol. Phys. **1974**, 27, 789–807.
51. T. Helgaker, M. Jaszuński, K. Ruud, Chem. Rev. **1999**, 99, 293–352.
52. S. Bloodworth, et al., Angew. Chem. Int. Ed. **2019**, 58, 5038–5043.
53. R. J. Kurland, B. R. McGarvey, J. Magn. Reson. **1970**, 2, 286–301.
54. W. Van den Heuvel, A. Soncini, J. Chem. Phys. **2013**, 138, 054113.
55. J. Vaara, S. A. Rouf, J. Mareš, J. Chem. Theory Comput. **2015**, 11, 4840–4849.
